# Dense hydrated Mg-silicates in diamond: Implications for transport of H_2_O into the mantle

**DOI:** 10.1126/sciadv.adl4306

**Published:** 2024-03-13

**Authors:** Luísa D. V. Carvalho, Thomas Stachel, Robert W. Luth, Andrew J. Locock, D. Graham Pearson, Matthew Steele-MacInnis, Richard A. Stern, Fabrizio Nestola, Ricardo Scholz, Tiago Jalowitzki, Reinhardt A. Fuck

**Affiliations:** ^1^ University of Alberta, Department of Earth and Atmospheric Sciences, 1-26 ESB, T6G 2E3 Edmonton, AB, Canada.; ^2^ Università di Padova, Dipartimento di Geoscienze, Via Gradenigo, 6-35131 Padova, Italy.; ^3^ Universidade Federal de Ouro Preto (UFOP), Escola de Minas, Departamento de Geologia, Campus Morro do Cruzeiro, 35400-000 Ouro Preto, MG, Brazil.; ^4^ Universidade de Brasília (UnB), Instituto de Geociências, Campus Universitário Darcy Ribeiro, 70910-900 Brasília, DF, Brazil.

## Abstract

Water in Earth’s upper mantle is a minor and yet critically important component that dictates mantle properties such as strength and melting behavior. Minerals with stoichiometric water, such as those of the humite group, are important yet poorly characterized potential reservoirs for volatiles in the upper mantle. Here, we report observation of hydroxyl members of the humite group as inclusions in mantle-derived diamond. Hydroxylchondrodite and hydroxylclinohumite were found coexisting with olivine, magnesiochromite, Mg-bearing calcite, dolomite, quartz, mica, and a djerfisherite-group mineral in a diamond from Brazil. The olivine is highly forsteritic (Mg# 97), with non–mantle-like oxygen isotope composition (δ^18^O +6.2‰), and is associated with fluid inclusions and hydrous minerals—features that could be inherited from a serpentinite protolith. Our results constitute direct evidence for the presence of deserpentinized peridotitic protoliths in subcratonic mantle keels, placing important constraints on the stability of hydrous phases in the mantle and the origin of diamond-forming fluids.

## Teaser

A metaserpentinite-derived diamond inclusion assemblage documents transport of surficial water into deep lithospheric mantle.

## INTRODUCTION

Carrier phases for water control the distribution of this key volatile in Earth’s mantle. Whereas the key hosts for mantle water in the transition zone are now well understood [e.g., (*1*–*3*)], the potential hosts for upper mantle water are many and varied (*4*, *5*), with little evidence for the existence of many contenders beyond phlogopite and amphibole. The discovery of hydrous minerals in upper mantle assemblages is thus important to enhance our understanding of upper mantle water distribution.

Subducting slabs are the main mechanism of transporting water and other volatiles back to Earth’s mantle because of the variable hydration of oceanic lithosphere (*4*), with minerals such as serpentine, mica, chlorite, apatite, and amphibole being the most commonly considered (*6*). If serpentine is a major constituent of altered oceanic lithosphere, the progressive dehydration of the down-going slab during prograde metamorphism is generally referred to as deserpentinization (*7*). Fluids released by this process play an important role in hydration, metasomatism, and partial melting of the overlying mantle wedge (*8*) and in controlling the physical properties of the slab (*4*). Experiments show that upon breakdown of the serpentine-group mineral antigorite, dense hydrous magnesium silicates (DHMSs) stabilize [see (*9*)]. Among DHMSs, members of the humite group, particularly hydroxylchondrodite Mg_5_(SiO_4_)_2_(OH,F)_2_ and hydroxylclinohumite Mg_9_(SiO_4_)_4_(OH,F)_2_, have been suggested to be important hosts for water in the upper mantle [e.g., (*10*)], but direct evidence has been lacking and these minerals are rarely considered. Humite-group minerals have a crystal structure closely resembling that of olivine (*11*) and can store several weight percent of structural water, in addition to fluorine (*12*). Natural occurrences of humite-group minerals are generally associated with ultramafic massifs [e.g., (*13*) and references therein], contact zones of skarns (*14*), or secondary minerals in kimberlites [discovery by Aoki *et al.* (*15*) as reinterpreted by Smith (*16*); (*17*, *18*)] and carbonatites (*19*). In diamond, clinohumite (*20*) and chondrodite (*21*) have only been documented as secondary phases with poorly defined grain boundaries, occurring within polycrystalline precipitates from nanometer-sized original fluid inclusions in diamonds from Siberia.

Here, we report the occurrence of two humite-group minerals included within a diamond from Brazil, which sheds light on the downward transport of water into the diamond stability field (>140 km) and the role of hydrated slabs in forming cratonic lithosphere. In particular, we provide direct evidence of humite-group minerals as principal hosts of water in the observed mineral assemblage.

## RESULTS

**Fig. 1.**
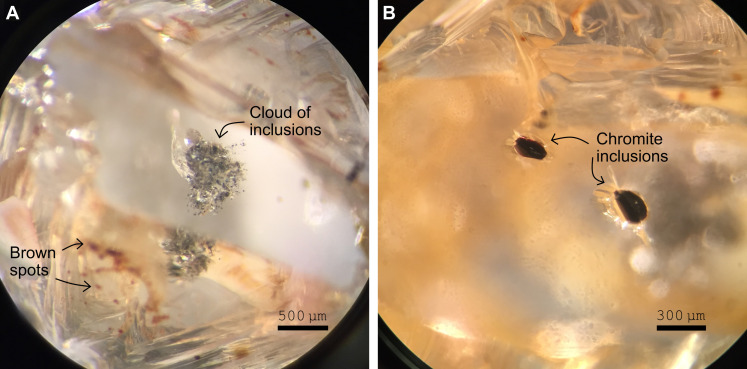
Mineral inclusions in the studied diamond. (**A**) Cloud of small and larger inclusions in the center of the studied octahedral diamond crystal. Multiple mineral inclusions were recovered from this cloud after diamond breakage. (**B**) Large magnesiochromite inclusions at the edge of the same diamond. In (A) and (B), the inclusions are seen through faces polished in {110} (dodecahedral planes). Brown spots observed on the original diamond surface were originally green but changed color due to heating associated with polishing.

The studied diamond had an octahedral external morphology and weighed 1.3 ct. It contained a set of inclusions scattered across an area of about 500 μm^2^ in the center of the crystal (Fig. 1A). Five magnesiochromite inclusions, measuring between 100 and 250 μm in largest dimension, occurred close to the crystal edge (Fig. 1B). No fractures connect any of the inclusions to the diamond surface. The inclusions recovered from the central part of the crystal ranged in diameter from 10 to 100 μm and included olivine, magnesiochromite, hydroxylclinohumite, Mg-bearing calcite, dolomite, and quartz. Hydroxylchondrodite, mica, and a djerfisherite-group mineral were also found, as inclusions within the recovered olivine grains.

The diamond has high concentrations of nitrogen, predominantly in A centers (pairs of nitrogen atoms) substituting for carbon. An inclusion-bearing fragment from the inner part of the diamond has a nitrogen content of ~365 atomic parts per min (at.ppm), with 29 %B centers [four nitrogen atoms surrounding a vacancy; %B = 100N_B_/(N_A_ + N_B_)]. An inclusion-free cross section of the diamond showed nitrogen contents ranging from ~250 to 360 at.ppm, with 20 to 30 %B. A diamond fragment containing a magnesiochromite inclusion from the outer part of the diamond revealed that the proportion of B centers decreases toward the edge. Nitrogen contents and aggregation states vary from ~185 to 270 at.ppm and 6 to 12 %B around the magnesiochromite inclusion.

The three analyzed olivine grains have very high Mg# [100 Mg/(Mg + Fe)], ranging from 96.5 to 97.4, NiO contents from 0.29 to 0.34 wt %, and low MnO concentrations (0.04 to 0.06 wt %) (Table 1). The oxygen isotope composition (δ^18^O) of this olivine is +6.17 ± 0.14 (2σ).

**Table 1.** Chemical composition of studied mineral inclusions from Brazilian diamond. Average major element composition (wt %) and formula proportions of mineral inclusions analyzed by EPMA. Values below detection are omitted. The number of contributing points is given by *n*. Na_2_O was not found above detection. F was measured only for hydroxylchondrodite and mica; the oxygen equivalent of fluorine (O═F) was subtracted.

Mineral	Forsterite			Hydroxylchondrodite		Mica	Quartz	Mg-Chr	Mg-Chr
(*n*)	(3)	(3)	(2)	(4)	(1)	(1)	(3)	(3)	(6)
Size (μm)	100 × 80	75 × 75	75 × 50	20 × 7	5 × 4	7 × 4	60 × 30	40 × 25	>100
Sample	Olivine 1	Olivine 2	Olivine 3	Olivine 2	Olivine 3	Olivine 3	Quartz	Inner chr	Outer chr
SiO_2_	41.97	42.02	41.68	35.19	37.54	41.55	99.43		
TiO_2_				0.06			0.04	0.02	0.02
Al_2_O_3_	0.01					2.19	0.06	9.26	9.12
Cr_2_O_3_								60.23	63.09
FeO	3.49	2.61	2.76	0.76	0.71	1.26		10.90	10.84
Fe_2_O_3_						12.35		2.89	1.66
NiO	0.34	0.29	0.33		0.09	0.04		0.11	0.11
MnO	0.06	0.04	0.04			0.02		0.19	0.16
MgO	53.92	54.58	54.54	58.52	58.61	29.25		14.10	14.54
CaO	0.01	0.02				0.03	0.01		
K_2_O						9.56			
F				4.73	2.78				
O═F				−1.99	−1.17				
H_2_O_calc_				3.03	3.97	4.14			
Total	99.83	99.62	99.38	100.35	102.57	100.38	99.60	97.41	99.54
Cations	3.00	3.00	3.00	8.15	8.50	10.00	1.00	3.00	3.00
Si	1.002	1.001	0.995	2.001	2.098	3.012	0.998		
Ti				0.002			0.000	0.001	0.000
Al	0.000					0.187	0.001	0.359	0.347
Cr								1.568	1.612
Fe^2+^	0.070	0.052	0.055	0.036	0.033	0.076		0.300	0.293
Fe^3+^						0.674		0.072	0.040
Ni	0.007	0.006	0.006		0.004	0.002		0.003	0.003
Mn	0.001	0.001	0.001			0.001		0.005	0.004
Mg	1.919	1.939	1.942	4.959	4.883	3.161		0.692	0.700
Ca	0.000	0.000		0.001		0.002	0.000		
K						0.884			
F				0.851	0.492				
O	4.002	4.001	3.996	9.153	9.611	8.000	1.999	4.000	4.000
H				1.149	1.481	2.000			

**Fig. 2.**
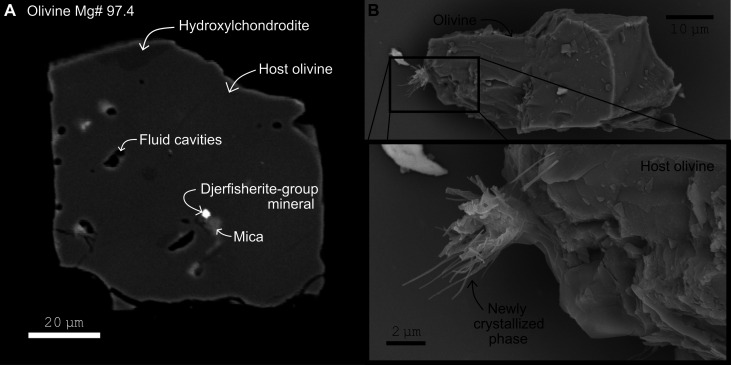
Diamond inclusion olivine showing a complex assemblage. (**A**) Backscattered electron image of an olivine inclusion (#2) that itself includes hydroxylchondrodite, a djerfisherite-group mineral, mica, and fluid (which escaped at surface to leave behind empty cavities). (**B**) Secondary electron image of an olivine inclusion (#4) showing a phase crystallized after release from the host diamond during escape of entrapped fluids.

The forsteritic olivines contained inclusions of hydrous phases identified as hydroxylchondrodite and mica (Fig. 2A and Table 1). The mica included in olivine grain 3 is magnesian with no fluorine detected (limit of detection = 0.03 wt %) and a H_2_O content of 4.1 wt %, calculated on the basis of stoichiometry. On the basis of its calculated mineral formula, K_0.88_Mg_3.16_Fe^2+^_0.08_(Si_3.01_Al_0.19_Fe^3+^_0.67_)O_10_(OH)_2_, it is identified as tetraferriphlogopite (Fe^3+^ calculated on the basis of eight cations aside from the hydroxyl group, based on a total of 12 oxygen; the excess Mg is probably a result of secondary fluorescence from the enclosing olivine).

**Fig. 3.**
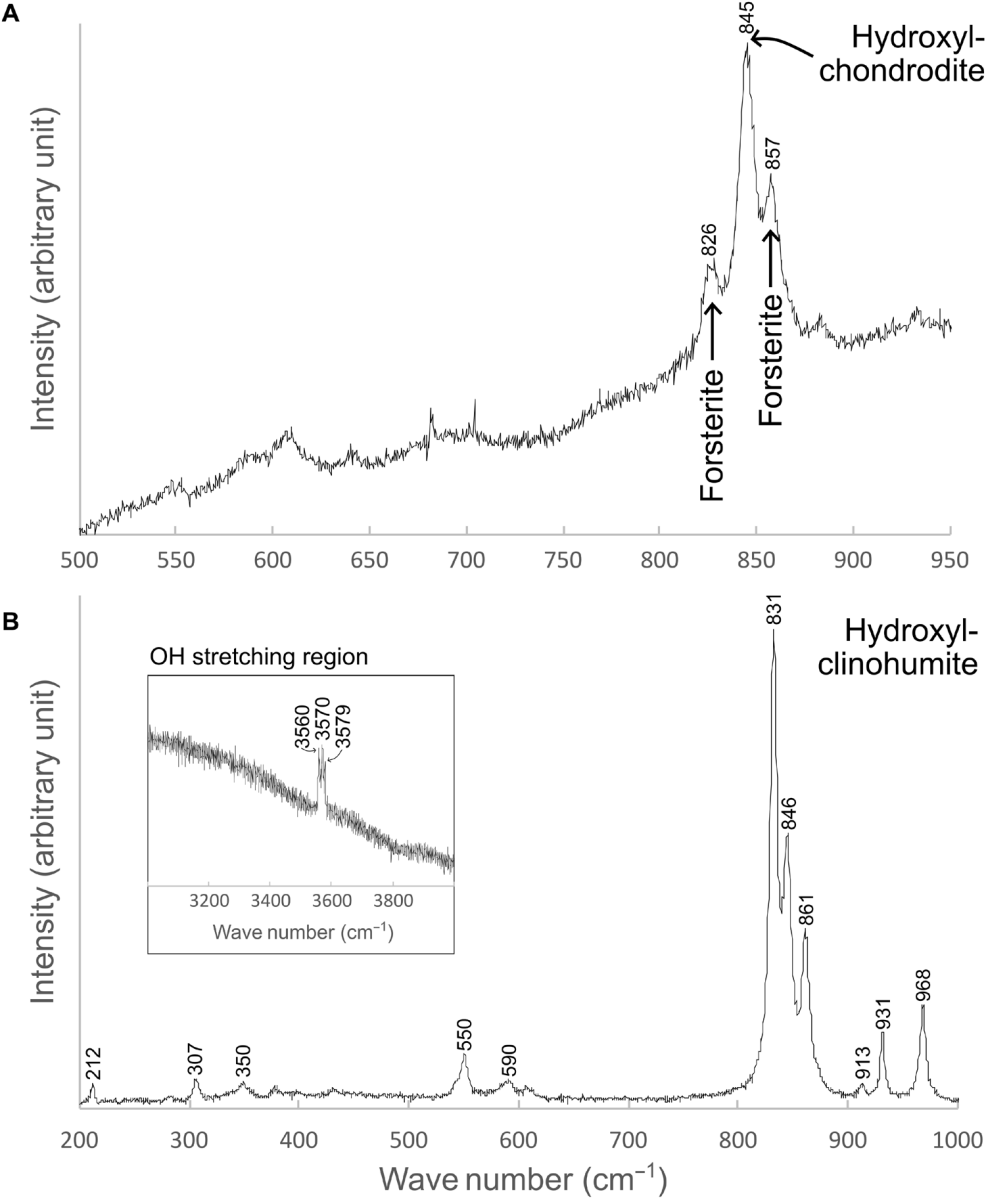
Raman spectra of humite minerals included in diamond in this study. (**A**) Raman spectrum of a hydroxylchondrodite included in an olivine inclusion in diamond. The peak at 845 cm^−1^ characterizes the main stretching vibrations of SiO_4_ units (*23*). The peaks at ~826 and 857 cm^−1^ offer a better match to the stretching vibrations of SiO_4_ units of the host olivine (*87*). (**B**) Raman spectrum of a hydroxylclinohumite, with inset showing the peaks at high wave numbers that correspond to the OH stretching region (*23*). The spectra are not smoothed or baseline-corrected.

Hydroxylchondrodite inclusions, identified by electron probe microanalysis and further confirmed by Raman spectroscopy (Fig. 3A) within two olivine grains, are very magnesian, with Mg# = 99.3. The average F content of hydroxylchondrodite in olivine 2 is 4.7 wt %, yielding an empirical formula of Mg_4.96_Fe^2+^_0.04_(SiO_4_)_2_F_0.85_(OH)_1.15_.

A K-Fe-Ni sulfide with minor Cl occurring as an inclusion in olivine grain 2 (Fig. 2A) was identified in energy-dispersive x-ray spectroscopy (EDS) analysis and interpreted as a djerfisherite-group mineral (fig. S1). Djerfisherite is documented from kimberlites, mantle xenoliths, and as inclusions in diamond, but its stability at high pressure and temperature is poorly constrained [see (*22*) and references therein]. Its paragenesis usually reflects the interaction between K-Cl–rich fluid/melts and Fe-Ni-Cu sulfides (*22*). The crystal in this study was too small (~2 μm × 2 μm) for quantitative analysis.

A fourth olivine grain displayed evidence for the presence of fluid inclusions. After resting for 8 months at room temperature on a glass slide, we observed that needles grew out of the crystal surface (Fig. 2B). The newly formed mineral needles are interpreted to have crystallized from the escape of fluid trapped within the olivine. Analysis of the needles by EDS revealed the presence of C, O, K, and, likely, also Mg (fig. S2). On the basis of these observations, small cavities observed in the polished olivine grains are interpreted to represent opened fluid inclusions. This observation is further supported by the presence of mica at the rims of such cavities (Fig. 2A); the mica is probably a product of reaction between the K-bearing fluid and the host olivine.

Besides olivine and its included phases, a SiO_2_ phase and three magnesiochromite inclusions were released by crushing. The SiO_2_ phase is 99.4 wt % SiO_2_ and was confirmed to be quartz by Raman spectroscopy (fig. S3), likely retrogressed from primary coesite. Magnesiochromite grains recovered from the inner and outer parts of the diamond are of similar composition as each other (see Table 1).

A recovered inclusion measuring only ~10 μm could not be polished for chemical analysis but was examined via Raman spectroscopy. Triplets recognized at ~831, 846, and 861 cm^−1^ and ~3560, 3570, and 3579 cm^−1^ (Fig. 3B) offer a good match to hydroxyl-bearing clinohumite (*23*). Single-crystal x-ray diffraction confirmed the identity of this grain as clinohumite or hydroxylclinohumite (it is not possible to be certain as to the ratio of F to OH in this grain from the Raman or single-crystal x-ray diffraction methods; elsewhere in the article, we elect to refer to it as hydroxylclinohumite), with the monoclinic cell having the unit dimensions: *a* = 4.7389(7) Å, *b* = 10.2229(19) Å, *c* = 13.6423(21) Å, α = 100.800(15)°, *V* = 649.20(18) Å^3^ (using the setting commonly reported for clinohumite in literature). Other mineral phases identified by Raman spectroscopy include Mg-bearing calcite and dolomite (fig. S3).

## DISCUSSION

High-Mg# olivine is generally associated with highly depleted, harzburgitic/dunitic diamond substrates (*24*, *25*). Extreme Mg# (>96) in primary (i.e., not retrogressed from higher pressure phases) olivine inclusions in diamonds, however, were previously recorded only twice, in both cases as Mg# 96.6 (*26*, *27*). The high-Mg# olivine reported by Jaques *et al.* (*26*) coexists with garnet and clinopyroxene in a diamond from Ellendale, Kimberley Craton, Australia. A high*-*Mg# olivine in a diamond from Wesselton, Kaapvaal Craton, South Africa, coexists with a magnesiochromite (*27*). All other occurrences of olivine inclusions with Mg# > 96, including a pure forsterite [Mg# 99.9; (*28*)], were found together with ferropericlase in sublithospheric diamonds (*n* = 3). In those cases, olivine was interpreted as either retrogressed ringwoodite (*28*) or the product of reequilibration of lower mantle phases in the upper mantle (*29*, *30*).

In contrast to the high-Mg# olivines previously reported as inclusions in diamonds, the inclusion association in our study is unusual. Olivine with exceptionally high Mg# (Fo_96–98_) coexisting with humite-group minerals, mica, spinel, quartz, carbonates, and sulfides is characteristic of ultramafic assemblages that underwent deserpentinization during prograde metamorphism [see (*13*, *31*–*34*) and references therein]. The highly magnesian character of serpentine minerals results from the redox processes associated with serpentinization, which causes iron oxides, sulfides, and native iron alloys to precipitate (*35*). Olivine that forms by the dehydration breakdown of antigorite inherits this high-Mg# characteristic. This breakdown also liberates large amounts of water [up to 13 wt %; (*8*, *36*, *37*)]. Beyond the antigorite stability field, the remaining water budget will be hosted by dense hydrous magnesium phases (*38*–*40*), namely, the humite-group minerals (*12*). Another characteristic inherited from serpentinite protoliths is the enrichment in halogens (*41*–*44*), consistent with the presence of F- and Cl-bearing minerals as inclusions in the diamond in this study.

Given the above observations, a serpentinite protolith is very likely for the mineral assemblage observed in this study. The high δ^18^O value determined from olivine grain 2 (+6.2‰) supports this interpretation. Serpentinization of ultramafic rocks occurs by exchange with seawater at relatively low temperatures (*45*, *46*), resulting in the highly variable (~+2 to +8‰) oxygen isotope signatures observed for serpentinite protoliths (*47*–*49*). In contrast, the peridotitic mantle comprising cratonic lithosphere is extremely restricted in its δ^18^O composition [around +5.5‰; (*50*, *51*)], which allows the use of oxygen isotopes as tracers of crustal recycling within the lithospheric root (*52*–*54*).

Subduction transports altered oceanic crust/lithosphere to depths of the diamond stability field (*55*, *56*). The subduction association of, likely all, eclogitic diamond substrates is widely accepted (*57*–*60*). The peridotitic subcratonic lithospheric mantle, on the other hand, lacks any crustal signatures (*51*), having an origin understood as residues of large degrees of melt extraction (*61*–*63*). The depleted signature of mantle peridotites is reflected in the composition of peridotitic mineral inclusions in diamonds (*60*, *64*). A primary origin of cratonic peridotites, particularly the characteristic subcalcic garnets within them, from serpentinized oceanic peridotites was proposed by Schulze (*65*) but subsequently shown to be inconsistent with the overwhelmingly mantle-like oxygen isotope composition of olivine from cratonic peridotites (*51*).

Diamond formation in a noncratonic setting, having occurred in metarodingites in subducted oceanic crust, was proposed by Davies *et al.* (*66*). Our diamond sample, however, has the characteristics (octahedral morphology, mild resorption, and moderate levels of nitrogen aggregation) that are typically associated with diamonds formed and stored in subcratonic lithospheric mantle (*25*, *67*). The presence of two types of humite-group minerals together with evidence of excess fluid, trapped as fluid inclusions, plus the elevated δ^18^O value of the high-Mg# olivine inclusions all combine to provide evidence for the presence of deserpentinized peridotitic diamond substrates in subcratonic mantle keels. Contrary to the suggestion of a widespread origin of cratonic peridotites as metaserpentinites (*65*), the otherwise general rarity of the ^18^O-enriched isotopic signature observed for olivine in our sample [see (*51*)] indicates that we observe evidence of a process that is uncommon in the formation of cratonic peridotites but may mark a horizon of unusually altered oceanic mantle lithosphere that became lodged in the lower portions of the mantle lithosphere during craton formation.

The occurrence of a deserpentinization-related peridotitic assemblage within a diamond places strong constraints on the stability of hydrous phases in the mantle. Deserpentinized rocks have been reported for conditions varying from contact (*68*), to regional (*7*), or ultrahigh-pressure metamorphism (*69*). For the mineral assemblage in this study, pressure and temperature (*P*-*T*) conditions within the diamond stability field are implicit. Typical cratonic conductive geotherms of 38 to 40 mW/m^2^ intersect the graphite-diamond transition at about 40 kbar and 900° to 1000°C (*70*, *71*), providing a minimum estimate for the upper *P*-*T* stability limit for the assemblage in this study, allowing an examination of the *P*-*T* stability of hydrous magnesium phases and coexisting minerals in the MgO ± SiO_2_ ± H_2_O (MSH) system.

### Implications for transport and storage of H_2_O in the upper mantle

The concept of humite-group minerals representing important reservoirs for volatiles in the upper mantle first emerged in the 1970s (*10*, *72*). Experimentally, these minerals were proven to be stable over a wide *P*-*T* range (*39*, *40*, *73*, *74*). The hydroxyl endmembers [(*74*) for hydroxylchondrodite; (*40*) for hydroxylclinohumite] were synthesized at *P*-*T* conditions >3 GPa and >700°C. In nature, however, hydroxylchondrodite and hydroxylclinohumite were first recognized associated with low-P contact metamorphism (*14*, *75*). Titanium-rich hydroxylchondrodite and hydroxylclinohumite commonly occur in deserpentinized assemblages [e.g., (*13*, *31*, *33*, *34*)]. To evaluate the extent to which the occurrence of the touching (=equilibrium) assemblage forsterite (Fo) + hydroxylchondrodite (Chn) + fluid (V; stands for “vapor” in previous works) observed in olivine grain 2 (Fig. 2A) is consistent with experimental work, we revisited the phase diagrams constructed by Yamamoto and Akimoto (*40*) and Wunder (*39*).

In the experiments of Yamamoto and Akimoto (*40*), Fo + Chn + V coexists with brucite (Brc) and hydroxylclinohumite (Chu) at *P*-*T* conditions of 2.9 GPa and 815°C, 4 GPa and 745°C, and 4.8 GPa and 730°C. Despite the observed assemblage, they excluded the assemblage from their phase diagram as the number of phases contradicts the phase rule. The experiments of Wunder (*39*) also do not predict a common stability for Fo + Chn + V. For *P*-*T* conditions in excess of ~10 GPa and 900°C, a field exists between Wunder’s invariant points III {Fo, phase-A [Mg_7_Si_2_O_8_(OH)_6_, ph-A], Chu, Chn} and VI {phase-B [Mg_10_Si_3_O_14_(OH)_4_], ph-A, Fo, Chn, periclase (Per)} where either Fo + Chn or Chn + V is stable but not both. In addition, Wunder (*39*) considers invariant points III and VI not well constrained.

**Fig. 4.**
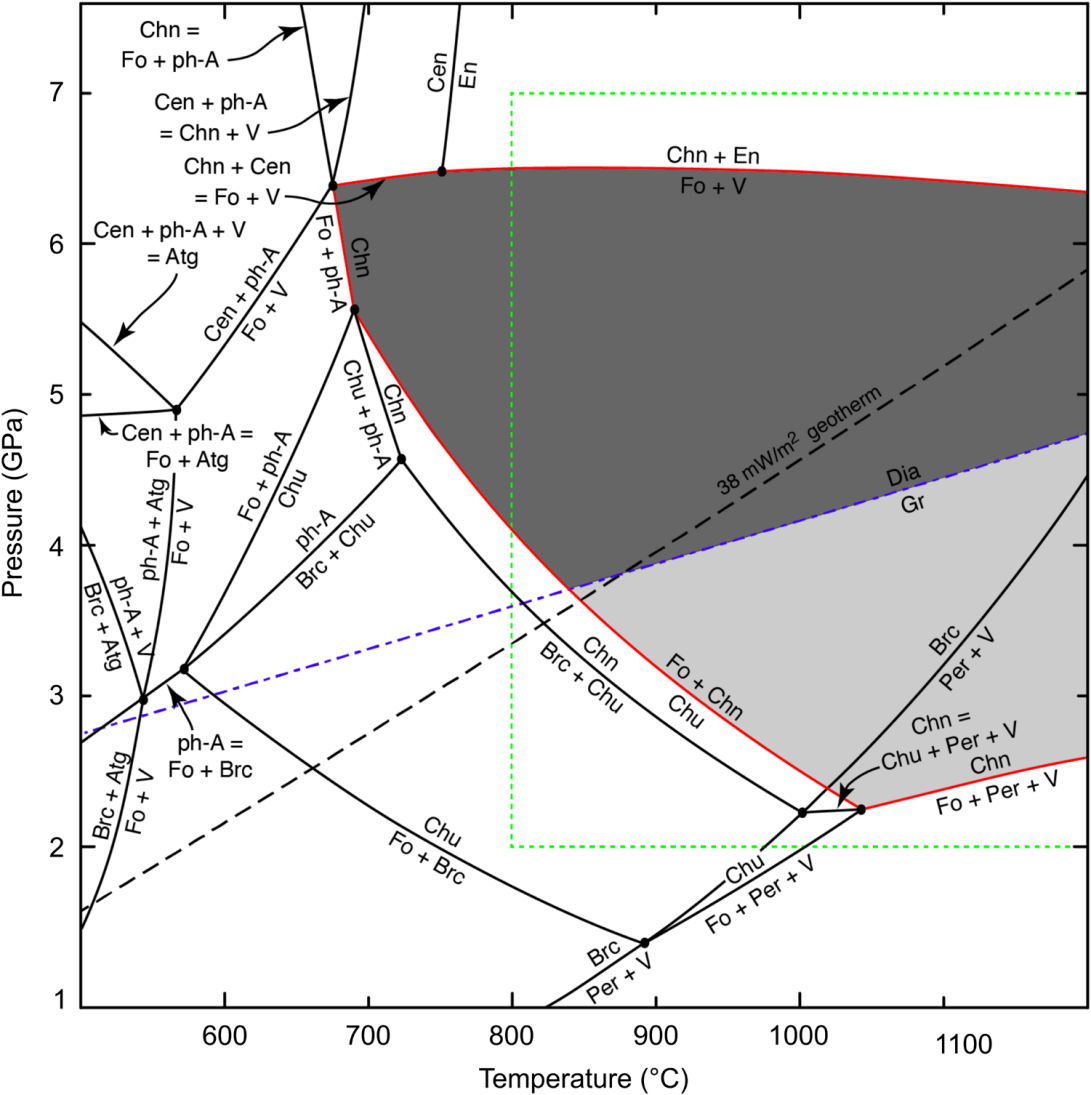
Phase equilibria showing the stability field for the diamond inclusion assemblage in this study. Calculated phase equilibria in the MSH system showing reactions involving DHMSs in the *P*-*T* region 1 to 7.5 GPa and 500° to 1200°C. The relevant reactions that constrain the coexistence of forsterite (Fo) + chondrodite (Chn) + fluid (V) are highlighted in red. The diamond-graphite boundary (*70*) and a 38 mW/m^2^ conductive model geotherm (*71*) are shown for reference. The stability field of Fo + Chn + V in the presence of diamond is shaded dark gray; the field for Fo + Chn + V in the presence of graphite is shaded light gray. The rectangle outlined by the green dashed lines is the *P*-*T* area shown in Fig. 5.

Given the uncertainties of the experimental data related to the *P*-*T* stability of the observed phase assemblage, we instead used the thermodynamic database of Holland and Powell (*76*) to calculate the *P*-*T* projections of reactions in the MSH system. We used the most recent database (ds634) and THERMOCALC version 3.50 for these calculations. The *P*-*T* projections and compatibility diagrams, as calculated by THERMOCALC, are shown in fig. S4. Phase relationships relevant to the stability of chondrodite are shown in Fig. 4. These calculated results are in stark contrast with previous experimental work, revealing a wide stability field for Fo + Chn + V (gray areas in Fig. 4). This field is bounded by the reactions Fo + V = Chn + enstatite (En), Chn = Fo + ph-A, Fo + Chn = Chu, and Chn = Fo + Per + V, highlighted in red (Fig. 4). Considering that the observed phase assemblage occurs in equilibrium with diamond, Fig. 4 also includes the diamond-graphite boundary after Day (*70*) and a 38 mW/m^2^ conductive model geotherm after Hasterok and Chapman (*71*). The calculated stability field for Fo-Chn-V extends into the diamond stability field along the entire range of typical cratonic geotherms (38 to 42 mW/m^2^).

Besides the large intrinsic uncertainties surrounding the phase equilibria calculated in the MSH system with THERMOCALC, two additional observations need to be considered: (i) the fluorine content (2.8 to 4.7 wt %) of the hydroxylchondrodite inclusions, which is known to have a strong effect on *P*-*T* stability (*41*, *77*), and (ii) the isolated presence of hydroxylclinohumite within the same diamond.

**Fig. 5.**
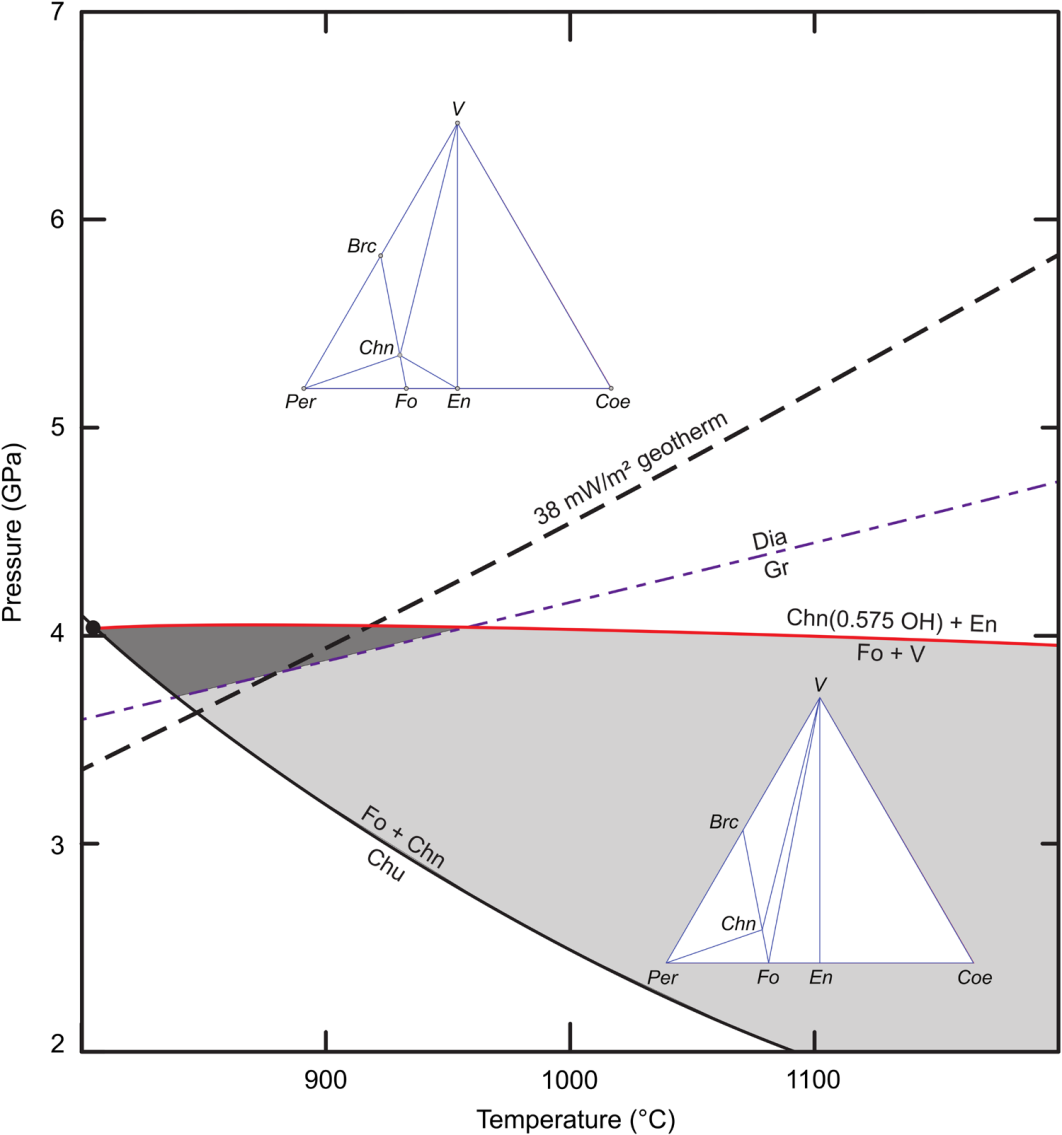
Phase equilibria considering the activity of fluorine. Subsection of the *P*-*T* space shown in Fig. 4 showing the shift of the forsterite (Fo) + fluid (V) = chondrodite (Chn) + enstatite (En) phase boundary to lower pressure in the presence of fluorine (see text for details). The more restricted stability field for Fo + Chn + V + diamond is highlighted in dark gray. The two ternary diagrams show the phase compatibilities for two of the fields in this *P*-*T* projection, indicating the change across the Chn + En = Fo + V reaction. The complete set of these compatibility diagrams is shown in fig. S4.

To assess the influence of fluorine on the stability field of Fo + Chn + V, we used the measured OH^−^/(OH^−^ + F^−^) ratio (0.575) of our chondrodite inclusions to recalculate the *P*-*T* positions of our reactions. Assuming that F partitions fairly equally into chondrodite and clinohumite, the hydroxyl activity in both minerals changes from 1^2^ to 0.575^2^. Figure 5 shows how the incorporation of F affects the *P*-*T* stability of our assemblage, assuming it to be in equilibrium: Whereas the main clinohumite breakdown reaction, limiting the low temperature side of this field, is not affected by OH or F content, the presence of F shifts the upper and lower pressure limits of the Fo + Chn + V stability downward. The shift by more than 2 GPa in the Fo + V = Chn + En reaction destabilizes our assemblage (Fo + Chn + V) for most of the diamond stability field within the cratonic mantle (e.g., along a 38 mW/m^2^ model geotherm). The assemblage Chn + Fo + En or Chn + V + En would be stable instead.

The calculated stability field of Fo + Chn + V + diamond in the presence of fluorine is limited to a restricted *P*-*T* range near the diamond-graphite boundary (Fig. 5). The new field is shifted toward the Fo + Chn = Chu reaction, and along this phase, boundary clinohumite coexists with Fo + Chn + V. Equilibrium between nontouching inclusions in diamonds is much more common than disequilibrium (*78*). If the full assemblage observed within the diamond in this study were in equilibrium, then formation near the diamond-graphite boundary along a 38 mW/m^2^ model geotherm would be implied. Given the peculiar assemblage and unusual mineral compositions (e.g., extreme Mg# of olivine), single-grain geothermometers [Al and Cr in olivine or Zn in spinel; (*79*–*81*)], which assume equilibration in compositionally “normal” garnet peridodites, cannot be applied with confidence. Diamond formation and storage at “cold” conditions in the cratonic mantle are in broad agreement with the low aggregation state of nitrogen in this diamond.

Although our calculated phase equilibria match well with the naturally occurring assemblage, including the stability of diamond, we must acknowledge that equilibrium between Fo + Chn + V ± Chu was not predicted on the basis of any published experiments. Our observations thus provide motivation to revisit these phase relationships at diamond-stable *P*-*T* conditions to calibrate clinohumite breakdown reactions. These experiments require the use of a rocking multi-anvil apparatus to avoid chemical segregation effects (*82*). Experiments in more complex chemical systems could constrain the effect of fluorine on shifting the phase fields in *P*-*T* space and allow assessment of the full phase assemblage observed here, including magnesiochromite and carbonate minerals.

This finding of hydroxylchondrodite and hydroxylclinohumite inside a diamond confirms concepts proposed five decades ago, which predicted the ability of humite-group minerals to store water and transport it into the deep upper mantle. The observation that a partially dehydrated metaserpentinite assemblage may become incorporated into cratonic lithosphere and act as a diamond substrate highlights the role that humite-group minerals may play during the subduction of hydrated oceanic lithosphere and the formation of diamond-forming fluids. The viability of water transport into the diamond stability field through humite-group minerals, followed by devolatilization, also lends strong support to a model (*60*, *83*) that links eclogitic diamond formation to flushing of subducting metabasaltic crust by serpentinite-derived hydrous fluids. The presence of dense hydrous Mg-silicates in diamond thus reaffirms that serpentine-derived subducted fluids play a key role in diamond formation processes.

## MATERIALS AND METHODS

The studied diamond was purchased from a diamond dealer in Minas Gerais, Brazil. It is of alluvial origin and hence of unknown location. The diamond in this study was broken using a steel crusher for the recovery of mineral inclusions. Diamond fragments containing inclusions from both the inner and outer zones as well as an inclusion-free section through the entire diamond were analyzed for their nitrogen concentration and aggregation states by micro–Fourier transform infrared (micro-FTIR) spectrometry. Spectra were collected in transmission mode over the range of 4000 to 650 cm^−1^ using a Thermo Fisher Scientific Nicolet iN10 MX in the De Beers Laboratory for Diamond Research at the University of Alberta. Analytical conditions included an aperture size of 100 μm^2^ and a spectral resolution of 2 cm^−1^, averaging 200 scans. Absorption spectra were recorded with a liquid nitrogen–cooled Mercury-Cadmium-Telluride (MCT) detector. The quantification of nitrogen in different aggregation states was accomplished following the procedure described in (*84*).

The mineral inclusions were analyzed by micro-Raman spectroscopy at the University of Alberta using a Horiba LabRam HR Evolution Raman microscope coupled to a 532-nm laser. The Raman microscope system includes an Olympus petrographic microscope. Spectra were acquired on unoriented rough or polished surfaces using a long working-distance ×100 magnification lens. Four accumulations over a 30-s acquisition time were undertaken to improve the signal-to-noise ratio, and the laser was used at 25% of total power (nominally 100 mW at the source). Measurements were performed at room temperature, and no spectral manipulations, such as baseline correction or smoothing, were undertaken.

The identification of hydroxylclinohumite was confirmed by single-crystal x-ray diffraction performed at the Department of Geosciences, University of Padova, using a SuperNova single-crystal diffractometer by Rigaku Oxford Diffraction, equipped with a Dectris Pilatus 200 K area detector and with a Mo Kα x-ray microsource. The sample-to-detector distance was set to 68 mm, and the operating conditions were 50 kV and 0.8 mA.

The concentrations of major and minor elements in olivine and its mineral inclusions, magnesiochromite, and quartz were acquired in several sessions on individually mounted and polished grains using a JEOL JXA-8900R electron probe microanalyzer (EPMA) equipped with five wavelength-dispersive spectrometers at the University of Alberta. Operating conditions were an accelerating voltage of 15 to 20 kV, a beam current of 20 to 30 nA, and a beam diameter of <1 to 2 μm. The x-ray lines, diffraction crystals, and standards used were Ti Kα, PET, rutile; Si Kα, TAP, quartz, pyrope, forsterite, enstatite; Fe Kα, LIF, fayalite; K Kα, PET, sanidine; Na Kα, TAP, albite; Ca Kα, PET, diopside, plagioclase; Al Kα, TAP, pyrope, plagioclase; Mn Kα, LIF, spessartine; Cr Kα, PET, Cr_2_O_3_; Ni Kα, LIF, nickel metal; Mg Kα, TAP, forsterite, enstatite, pyrope, periclase; F Kα, LDE1, MgF_2_. Peak and background counting times were 60 s for F and 30 s for all other elements. X-ray intensity data were reduced following Armstrong (*85*).

Some mineral phases, not mounted or polished, were imaged by scanning electron microscopy (SEM) using a Zeiss Sigma 300 VP Field Emission SEM coupled to a Bruker EDS system at the University of Alberta. Chemical composition was qualitatively assessed using an accelerating voltage of 15 kV. Before analysis, the grains were coated with 10-nm-thick gold.

The oxygen isotope (^18^O/^16^O) composition of one olivine grain was analyzed using a Cameca IMS 1280 multicollector ion microprobe at the Canadian Centre for Isotopic Microanalysis, University of Alberta. A ^133^Cs^+^ primary beam was operated with an impact energy of 20 keV and beam current of ~2.0 nA. The analytical methods are detailed by Ickert and Stern (*86*). In the present study, the instrumental mass fractionation calibration for olivine was determined by repeated concurrent analyses of reference materials (RMs). A third-order polynomial calibration curve was determined using four olivine RMs to model the systematic matrix effect of Mg# [100 Mg/(Mg + Fe)] on ^18^O^−^/^16^O^−^. The olivine RMs were S0013D (anchor RM, δ^18^O_VSMOW_ = +5.26‰, Mg# = 92.7), S0103 (δ^18^O_VSMOW_ = +5.2‰, Mg# = 73), S0159A (δ^18^O_VSMOW_ = +6.93‰, Mg# = 50.2), and S0211 (δ^18^O_VSMOW_ = +3.33‰, Mg# = 0.0). Repeat analysis of S0013D olivine (*n* = 11) yielded an SD of 0.04‰. For the single olivine in this study, the Mg# = 97.4 yields a matrix correction of only −0.17‰ relative to the uncorrected value when normalized to S0013D with a similar Mg#. The 95% confidence uncertainty estimates for δ^18^O_VSMOW_ for olivine unknowns average ±0.25‰.

## Supplementary Materials

### This PDF file includes:

Figs. S1 to S4 References

## References

[1] F. Nestola, J. R. Smyth, Diamonds and water in the deep Earth: A new scenario. Int. Geol. Rev. 58, 263–276 (2016).

[2] D. G. Pearson, F. E. Brenker, F. Nestola, J. McNeill, L. Nasdala, M. T. Hutchison, S. Matveev, K. Mather, G. Silversmit, S. Schmitz, B. Vekemans, L. Vincze, Hydrous mantle transition zone indicated by ringwoodite included within diamond. Nature 507, 221–224 (2014). 24622201 10.1038/nature13080

[3] J. R. Smyth, β-Mg_2_SiO_4_: A potential host for water in the mantle? Am. Min. 72, 1051–1055 (1987).

[4] M. Faccenda, Water in the slab: A trilogy. Tectonophysics 614, 1–30 (2014).

[5] A. B. Thompson, Water in the Earth’s upper mantle. Nature 358, 295–302 (1992).

[6] M. W. Schmidt, S. Poli, Devolatilization during subduction, in *Treatise on Geochemistry (Second Edition)*, H. D. Holland, K. K. Turekian, Eds. (Elsevier, 2014), pp. 669–701.

[7] J. A. Vance, M. A. Dungan, Formation of peridotites by deserpentinization in the Darrington and Sultan areas, Cascade Mountains, Washington. Geol. Soc. Am. Bull. 88, 1497 (1977).

[8] P. Ulmer, V. Trommsdorff, Serpentine stability to mantle depths and subduction-related magmatism. Science 268, 858–861 (1995). 17792181 10.1126/science.268.5212.858

[9] D. J. Frost, The stability of hydrous mantle phases. Rev. Mineral. Geochem. 62, 243–271 (2006).

[10] S-I. Akimoto, K. Yamamoto, K. Aoki, Hydroxyl-clinohumite and hydroxyl-chondrodite: Possible H_2_O-bearing minerals in the upper mantle, in *High-Pressure Research*, M.H. Manghnani, S-I. Akimoto, Eds. (Academic Press, 1977), pp. 163–172.

[11] P. H. Ribbe, G. V. Gibbs, N. W. Jones, Cation and anion substitutions in the humite minerals. Mineral. Mag. J. Mineral. Soc. 36, 966–975 (1968).

[12] N. W. Jones, P. H. Ribbe, G. V. Gibbs, Crystal chemistry of the humite minerals. Am. Min. 54, 391–411 (1969).

[13] J. M. Guotana, T. Morishita, I. Nishio, A. Tamura, T. Mizukami, K. Tani, Y. Harigane, K. Szilas, D. G. Pearson, Deserpentinization and high-pressure (eclogite-facies) metamorphic features in the Eoarchean ultramafic body from Isua, Greenland. Geosci. Front. 13, 101298 (2022).

[14] I. V. Pekov, E. I. Gerasimova, N. V. Chukanov, Y. K. Kabalov, N. V. Zubkova, A. E. Zadov, V. O. Yapaskurt, V. M. Gekimyants, D. Y. Pushcharovskii, Hydroxylchondrodite Mg_5_(SiO_4_)_2_(OH)_2_: A new mineral of the humite group and its crystal structure. Doklady Earth. Sci. 436, 230–236 (2011).

[15] K. Aoki, K. Fujino, M. Akaogi, Titanochondrodite and titanoclinohumite derived from the upper mantle in the Buell Park Kimberlite, Arizona, USA. USA. *Contrib. Mineral. Petrol.* 56, 243–253 (1976).

[16] D. Smith, Titanochondrodite and titanoclinohumite derived from the upper mantle in the Buell Park kimberlite, Arizona, USA. Contrib. Mineral. Petrol. 61, 213–215 (1977).

[17] A. Abersteiner, V. S. Kamenetsky, M. Kamenetsky, K. Goemann, K. Ehrig, T. Rodemann, Significance of halogens (F, Cl) in kimberlite melts: Insights from mineralogy and melt inclusions in the Roger pipe (Ekati, Canada). Chem. Geol. 478, 148–163 (2018).

[18] A. V. Golovin, V. V. Sharygin, N. P. Pokhilenko, Melt inclusions in olivine phenocrysts in unaltered kimberlites from the Udachnaya-East pipe, Yakutia: Some aspects of kimberlite magma evolution during late crystallization stages. Petrology 15, 168–183 (2007).

[19] R. H. Mitchell, Manganoan magnesian ilmenite and titanian clinohumite from the Jacupiranga carbonatite, Sao Paulo Brazil. Am. Min. 63, 544–547 (1978).

[20] A. M. Logvinova, R. Wirth, A. A. Tomilenko, V. P. Afanas’ev, N. V. Sobolev, The phase composition of crystal-fluid nanoinclusions in alluvial diamonds in the northeastern Siberian Platform. Russ. Geol. Geophys. 52, 1286–1297 (2011).

[21] A. M. Logvinova, I. S. Sharygin, Second natural occurrence of KFeS_2_ (Hanswilkeite): An inclusion in diamond from the Udachnaya kimberlite pipe (Siberian Craton, Yakutia). Minerals 13, 874 (2023).

[22] A. Abersteiner, V. S. Kamenetsky, K. Goemann, A. V. Golovin, I. S. Sharygin, A. Giuliani, T. Rodemann, Z. V. Spetsius, M. Kamenetsky, Djerfisherite in kimberlites and their xenoliths: Implications for kimberlite melt evolution. Contrib. Mineral. Petrol. 174, 8 (2019).

[23] R. L. Frost, S. J. Palmer, J. M. Bouzaid, B. J. Reddy, A Raman spectroscopic study of humite minerals. J. Raman Spectrosc. 38, 68–77 (2007).

[24] F. R. Boyd, Compositional distinction between oceanic and cratonic lithosphere. Earth Planet. Sci. Lett. 96, 15–26 (1989).

[25] T. Stachel, J. W. Harris, The origin of cratonic diamonds; constraints from mineral inclusions. Ore Geol. Rev. 34, 5–32 (2008).

[26] A. L. Jaques, A. E. Hall, J. W. Sheraton, C. B. Smith, S. S. Sun, R. M. Drew, C. Foudoulis, K. Ellingsen, Composition of crystalline inclusions and C-isotopic composition of Argyle and Ellendale diamonds, in *Kimberlites and Related Rocks*, J. Ross, A. L. Jaques, J. Ferguson, D. H. Green, O. S. Y. Reilly, R. V Danchin, A. J. A. Janse, Eds. (Geological Society of Australia, 1989), vols. 14; 2, pp. 966–989.

[27] T. Stachel, Diamond Inclusion Database. Borealis. V2 (2021); 10.7939/DVN/EJUE1G.

[28] F. Nestola, M. E. Regier, R. W. Luth, D. G. Pearson, T. Stachel, C. McCammon, M. D. Wenz, S. D. Jacobsen, C. Anzolini, L. Bindi, J. W. Harris, Extreme redox variations in a superdeep diamond from a subducted slab. Nature 613, 85–89 (2023). 36600063 10.1038/s41586-022-05392-8

[29] R. M. Davies, W. L. Griffin, S. Y. O’Reilly, B. J. Doyle, Mineral inclusions and geochemical characteristics of microdiamonds from the DO27, A154, A21, A418, DO18, DD17 and Ranch Lake kimberlites at Lac de Gras, Slave Craton, Canada. Lithos 77, 39–55 (2004).

[30] T. Stachel, J. W. Harris, G. P. Brey, W. Joswig, Kankan diamonds (Guinea) II: Lower mantle inclusion parageneses. Contrib. Mineral. Petrol. 140, 16–27 (2000).

[31] J. M. González-Jiménez, G. Plissart, L. N. Garrido, J. A. Padrón-Navarta, T. Aiglsperger, R. Romero, C. Marchesi, A. J. Moreno-Abril, M. Reich, F. Barra, D. Morata, Titanian clinohumite and chondrodite in antigorite serpentinites from Central Chile: Evidence for deep and cold subduction. Eur. J. Mineral. 29, 959–970 (2018).

[32] K. H. Hattori, S. Guillot, Geochemical character of serpentinites associated with high- to ultrahigh-pressure metamorphic rocks in the Alps, Cuba, and the Himalayas: Recycling of elements in subduction zones. Geochem. Geophys. Geosyst. 8, Q09010 (2007).

[33] P. Luoni, G. Rebay, M. I. Spalla, D. Zanoni, UHP Ti-chondrodite in the Zermatt-Saas serpentinite: Constraints on a new tectonic scenario. Am. Min. 103, 1002–1005 (2018).

[34] T. Shen, J. Hermann, L. Zhang, Z. Lü, J. A. Padrón-Navarta, B. Xia, T. Bader, UHP metamorphism documented in Ti-chondrodite- and Ti-clinohumite-bearing serpentinized ultramafic rocks from Chinese Southwestern Tianshan. J. Petrol. 56, 1425–1458 (2015).

[35] B. R. Frost, On the stability of sulfides, oxides, and native metals in serpentinite. J. Petrol. 26, 31–63 (1985).

[36] B. W. Evans, Control of the products of serpentinization by the Fe^2+^Mg^−1^ exchange potential of olivine and orthopyroxene. J. Petrol. 49, 1873–1887 (2008).

[37] B. Wunder, W. Schreyer, Antigorite: High-pressure stability in the system MgO-SiO_2_-H_2_O (MSH). Lithos 41, 213–227 (1997).

[38] M. Akaogi, S.-I. Akimoto, High-pressure stability of a dense hydrous magnesian silicate Mg_23_Si_8_O_42_H_6_ and some geophysical implications. J. Geophys. Res. 85, 6944–6948 (1980).

[39] B. Wunder, Equilibrium experiments in the system MgO-SiO_2_-H_2_O (MSH): Stability fields of clinohumite-OH [Mg_9_Si_4_O_16_ (OH)_2_], chondrodite-OH [Mg_5_Si_2_O_8_ (OH)_2_] and phase A (Mg_7_Si_2_O_8_(OH)_6_). Contrib. Mineral. Petrol. 132, 111–120 (1998).

[40] K. Yamamoto, S.-I. Akimoto, The system MgO-SiO_2_-H_2_O at high pressures and temperatures; stability field for hydroxyl-chondrodite, hydroxyl-clinohumite and 10 A o-phase. Am. J. Sci. 277, 288–312 (1977).

[41] S. Flemetakis, C. Tiraboschi, A. Rohrbach, J. Berndt, S. Klemme, The stability of antigorite in subduction zones revisited: The effect of F on antigorite stability and its breakdown reactions at high pressures and high temperatures, with implications for the geochemical cycles of halogens. Contrib. Mineral. Petrol. 177, 70 (2022).

[42] T. John, M. Scambelluri, M. Frische, J. D. Barnes, W. Bach, Dehydration of subducting serpentinite: Implications for halogen mobility in subduction zones and the deep halogen cycle. Earth Planet. Sci. Lett. 308, 65–76 (2011).

[43] M. Scambelluri, E. Cannaò, M. Gilio, The water and fluid-mobile element cycles during serpentinite subduction. A review. A review. *Eur. J. Mineral*. 31, 405–428 (2019).

[44] G. Segee-Wright, J. D. Barnes, J. C. Lassiter, D. J. Holmes, G. M. Beaudoin, R. Chatterjee, D. F. Stockli, J. E. Hoffmann, T. John, Halogen enrichment in the North American lithospheric mantle from the dehydration of the Farallon plate. Geochim. Cosmochim. Acta 348, 187–205 (2023).

[45] W. Bach, C. J. Garrido, H. Paulick, J. Harvey, M. Rosner, Seawater-peridotite interactions: First insights from ODP Leg 209, MAR 15°N. Geochem. Geophys. Geosyst. 5, Q09F26 (2004).

[46] B. W. Evans, K. Hattori, A. Baronnet, Serpentinite: What, why, where? Elements 9, 99–106 (2013).

[47] R. T. Gregory, H. P. Taylor, An oxygen isotope profile in a section of Cretaceous oceanic crust, Samail Ophiolite, Oman: Evidence for δ^18^O buffering of the oceans by deep (>5 km) seawater-hydrothermal circulation at mid-ocean ridges. J. Geophys. Res. Solid Earth 86, 2737–2755 (1981).

[48] M. Johnson, M. A. Dungan, J. A. Vance, Stable isotope compositions of olivine and dolomite in peridotites formed by deserpentinization, Darrington area, North Cascades, Washington. Geochim. Cosmochim. Acta 41, 431–435 (1977).

[49] A. P. Nutman, M. R. Scicchitano, C. R. L. Friend, V. C. Bennett, A. R. Chivas, Isua (Greenland) ~3700 Ma meta-serpentinite olivine Mg# and δ^18^O signatures show connection between the early mantle and hydrosphere: Geodynamic implications. Precambrian Res. 361, 106249 (2021).

[50] D. Mattey, D. Lowry, C. Macpherson, Oxygen isotope composition of mantle peridotite. Earth Planet. Sci. Lett. 128, 231–241 (1994).

[51] M. Regier, A. Miškovic, R. B. Ickert, D. G. Pearson, T. Stachel, R. A. Stern, M. Kopylova, An oxygen isotope test for the origin of Archean mantle roots. Geochem. Perspect. Lett. 9, 6–10 (2018).

[52] R. B. Ickert, T. Stachel, R. A. Stern, J. W. Harris, Diamond from recycled crustal carbon documented by coupled δ^18^O–δ^13^C measurements of diamonds and theirinclusions. Earth Planet. Sci. Lett. 364, 85–97 (2013).

[53] N. M. Korolev, A. E. Melnik, X.-H. Li, S. G. Skublov, The oxygen isotope composition of mantle eclogites as a proxy of their origin and evolution: A review. Earth Sci. Rev. 185, 288–300 (2018).

[54] D. J. Schulze, B. Harte, J. W. Valley, J. M. Brenan, D. M. D. R. Channer, Extreme crustal oxygen isotope signatures preserved in coesite in diamond. Nature 423, 68–70 (2003). 12721625 10.1038/nature01615

[55] S. E. Kesson, A. E. Ringwood, Slab-mantle interactions; III, The genesis of diamonds, in *International Congress of Geochemistry and Cosmochemistry*, Y. Bottinga, Ed. (Elsevier, 1988), vols. 70; 1–2, p. 52.

[56] K. Li, L. Li, D. G. Pearson, T. Stachel, Diamond isotope compositions indicate altered igneous oceanic crust dominates deep carbon recycling. Earth Planet. Sci. Lett. 516, 190–201 (2019).

[57] S. Aulbach, D. E. Jacob, Major- and trace-elements in cratonic mantle eclogites and pyroxenites reveal heterogeneous sources and metamorphic processing of low-pressure protoliths. Lithos 262, 586–605 (2016).

[58] H. H. Helmstaedt, D. J. Schulze, Southern African kimberlites and their mantle sample: Implications for Archean tectonics and lithosphere evolution, in *Kimberlites and Related Rocks*, J. Ross, Ed. (Blackwell, 1989), pp. 358–368.

[59] D. E. Jacob, Nature and origin of eclogite xenoliths from kimberlites. Lithos 77, 295–316 (2004).

[60] T. Stachel, S. Aulbach, J. W. Harris, Mineral Inclusions in lithospheric diamonds. Rev. Mineral. Geochem. 88, 307–391 (2022).

[61] F. R. Boyd, J. J. Gurney, Diamonds and the African lithosphere. Science 232, 472–477 (1986). 17743571 10.1126/science.232.4749.472

[62] D. G. Pearson, J. M. Scott, J. Liu, A. Schaeffer, L. H. Wang, J. van Hunen, K. Szilas, T. Chacko, P. B. Kelemen, Deep continental roots and cratons. Nature 596, 199–210 (2021). 34381239 10.1038/s41586-021-03600-5

[63] M. J. Walter, Melting residues of fertile peridotite and the origin of cratonic lithosphere, in *Mantle Petrology: Field Observations and High Pressure Experimentation: A Tribute to Francis R. (Joe) Boyd*, Y. Fei, C. M. Bertka, B. O. Mysen, Eds. (The Geochemical Society, 1999), vol. 6, pp. 225–239.

[64] J. J. Gurney, A correlation between garnets and diamonds in kimberlites, in *Kimberlite Occurrence and Origin: A Basis for Conceptual Models in Exploration*, J. E. Glover, P. G. Harris, Eds. (University of Western Australia, 1984), vol. 8, pp. 143–166.

[65] D. J. Schulze, Do peridotite-suite diamonds form in subducted serpentinites?, in *Fourth International Kimberlite Conference*, C. B. Smith, Ed. (Geological Society of Australia, 1986), vol. 16, pp. 424–425.

[66] R. M. Davies, W. L. Griffin, S. Y. O’Reilly, A. S. Andrew, Unusual mineral inclusions and carbon isotopes of alluvial diamonds from Bingara, eastern Australia. Lithos 69, 51–66 (2003).

[67] J. W. Harris, K. V. Smit, Y. Fedortchouk, M. Moore, Morphology of monocrystalline diamond and its inclusions. Rev. Mineral. Geochem. 88, 119–166 (2022).

[68] S. Arai, Contact metamorphosed dunite-harzburgite complex in the Chugoku district, western Japan. Contrib. Mineral. Petrol. 52, 1–16 (1975).

[69] Y. Chen, K. Ye, S. Guo, T.-F. Wu, J.-B. Liu, Multistage metamorphism of garnet orthopyroxenites from the Maowu mafic–ultramafic complex, Dabieshan UHP terrane, eastern China. Int. Geol. Rev. 55, 1239–1260 (2013).

[70] H. W. Day, A revised diamond-graphite transition curve. Am. Min. 97, 52–62 (2012).

[71] D. Hasterok, D. S. Chapman, Heat production and geotherms for the continental lithosphere. Earth Planet. Sci. Lett. 307, 59–70 (2011).

[72] T. R. McGetchin, L. T. Silver, A. A. Chodos, Titanoclinohumite: A possible mineralogical site for water in the upper mantle. J. Geophys. Res. 75, 255–259 (1970).

[73] T. Komabayashi, S. Omori, S. Maruyama, Experimental and theoretical study of stability of dense hydrous magnesium silicates in the deep upper mantle. Phys. Earth Planet. Inter. 153, 191–209 (2005).

[74] K. Yamamoto, S.-I. Akimoto, High pressure and high temperature investigations in the system MgO SiO_2_ H_2_O. J. Solid State Chem. 9, 187–195 (1974).

[75] V. M. Gekimyants, E. V. Sokolova, E. M. Spiridonov, G. Ferraris, N. V. Chukanov, M. Prencipe, V. N. Avdonin, Y. A. Polenov, Hydroxylclinohumite Mg_9_(SiO_4_)_4_(OH,F)_2_—A new mineral of the humite group. Proc. Russ. Mineral. Soc. 128, 64–70 (1999).

[76] T. J. B. Holland, R. Powell, An improved and extended internally consistent thermodynamic dataset for phases of petrological interest, involving a new equation of state for solids. J. Metam. Geol. 29, 333–383 (2011).

[77] L. Hughes, A. Pawley, Fluorine partitioning between humite-group minerals and aqueous fluids: Implications for volatile storage in the upper mantle. Contrib. Mineral. Petrol. 174, 78 (2019).

[78] T. Stachel, R. W. Luth, Diamond formation: Where, when and how? Lithos 220-223, 200–220 (2015).

[79] Y. Bussweiler, G. P. Brey, D. G. Pearson, T. Stachel, R. A. Stern, M. F. Hardman, B. A. Kjarsgaard, S. E. Jackson, The aluminum-in-olivine thermometer for mantle peridotites—Experimental versus empirical calibration and potential applications. Lithos 272–273, 301–314 (2017).

[80] J. C. M. De Hoog, L. Gall, D. H. Cornell, Trace-element geochemistry of mantle olivine and application to mantle petrogenesis and geothermobarometry. Chem. Geol. 270, 196–215 (2010).

[81] C. G. Ryan, W. L. Griffin, N. J. Pearson, Garnet geotherms: Pressure-temperature data from Cr-pyrope garnet xenocrysts in volcanic rocks. J. Geophys. Res. Solid Earth 101, 5611–5625 (1996).

[82] M. W. Schmidt, P. Ulmer, A rocking multianvil: Elimination of chemical segregation in fluid-saturated high-pressure experiments. Geochim. Cosmochim. Acta 68, 1889–1899 (2004).

[83] S. Aulbach, T. Stachel, L. M. Heaman, J. A. Carlson, Microxenoliths from the Slave Craton: Archives of diamond formation along fluid conduits. Lithos 126, 419–434 (2011).

[84] T. Stachel, J. W. Harris, L. Hunt, K. Muehlenbachs, A. Kobussen, EIMF, Argyle diamonds: How subduction along the Kimberley craton edge generated the world’s biggest diamond deposit, in *Geoscience and Exploration of the Argyle, Bunder, Diavik, and Murowa Diamond Deposits*, Andy T. Davy, Chris B. Smith, Herwart Helmstaedt, A. Lynton Jaques, John J. Gurney, Eds. (Society of Economic Geologists, 2018), vol. 20.

[85] J. T. Armstrong, CITZAF: A package of correction programs for the quantitative electron micro beam x-ray analysis of thick polished materials, thin films, and particles. Microbeam Anal. 4, 177–200 (1995).

[86] R. B. Ickert, R. A. Stern, Matrix corrections and error analysis in high-precision SIMS ^18^O/^16^O measurements of Ca–Mg–Fe garnet. Geostand. Geoanal. Res. 37, 429–448 (2013).

[87] K. E. Kuebler, B. L. Jolliff, A. Wang, L. A. Haskin, Extracting olivine (Fo–Fa) compositions from Raman spectral peak positions. Geochim. Cosmochim. Acta 70, 6201–6222 (2006).

[88] M. L. Frezzotti, F. Tecce, A. Casagli, Raman spectroscopy for fluid inclusion analysis. J. Geochem. Explor. 112, 1–20 (2012).

[89] L. N. Warr, IMA–CNMNC approved mineral symbols. Mineral. Mag. 85, 291–320 (2021).

