# Supplementary material for: Dense hydrated Mg-silicates in diamond: Implications for transport of H2O into the mantle
Source: Sci Adv. 2024 Mar 13;10(11):eadl4306. doi: 10.1126/sciadv.adl4306 (PMC10936952; doi:10.1126/sciadv.adl4306)
Supplement: Supplementary file 1 — Figs. S1 to S4 References [file sciadv.adl4306_sm.pdf]

Science Advances
AAAS

Supplementary Materials for

Dense hydrated Mg-silicates in diamond: Implications for transport of H_2O into the mantle

Luísa D. V. Carvalho *et al.*

Corresponding author: Luísa D. V. Carvalho, luisa.carvalho@ualberta.ca

Sci. Adv. **10**, eadl4306 (2024)
DOI: 10.1126/sciadv.adl4306

This PDF file includes:

Figs. S1 to S4
References

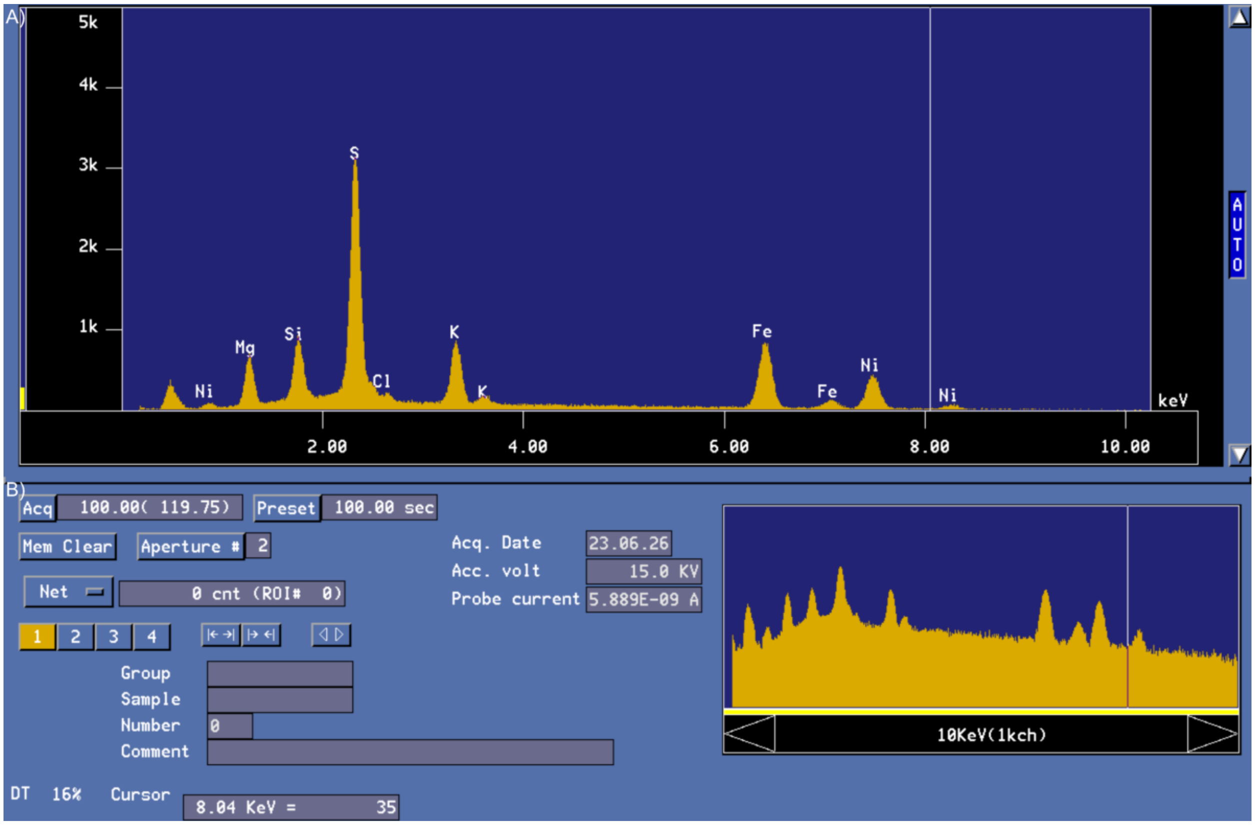

Fig. S1. Analyses by energy dispersive X-ray spectroscopy (EDS) of sulfide inclusion in olivine. A) Energy dispersive X-ray spectrum of a 2x2 µm inclusion in olivine grain 2 (bright spot in **Fig. 2A**). The Cl-bearing K-Fe-Ni sulfide is interpreted as a djerfisherite-group mineral. Si and Mg peaks are very likely from the olivine host. In **B)** (bottom) analytical conditions are indicated and the spectrum is shown in logarithmic scale.

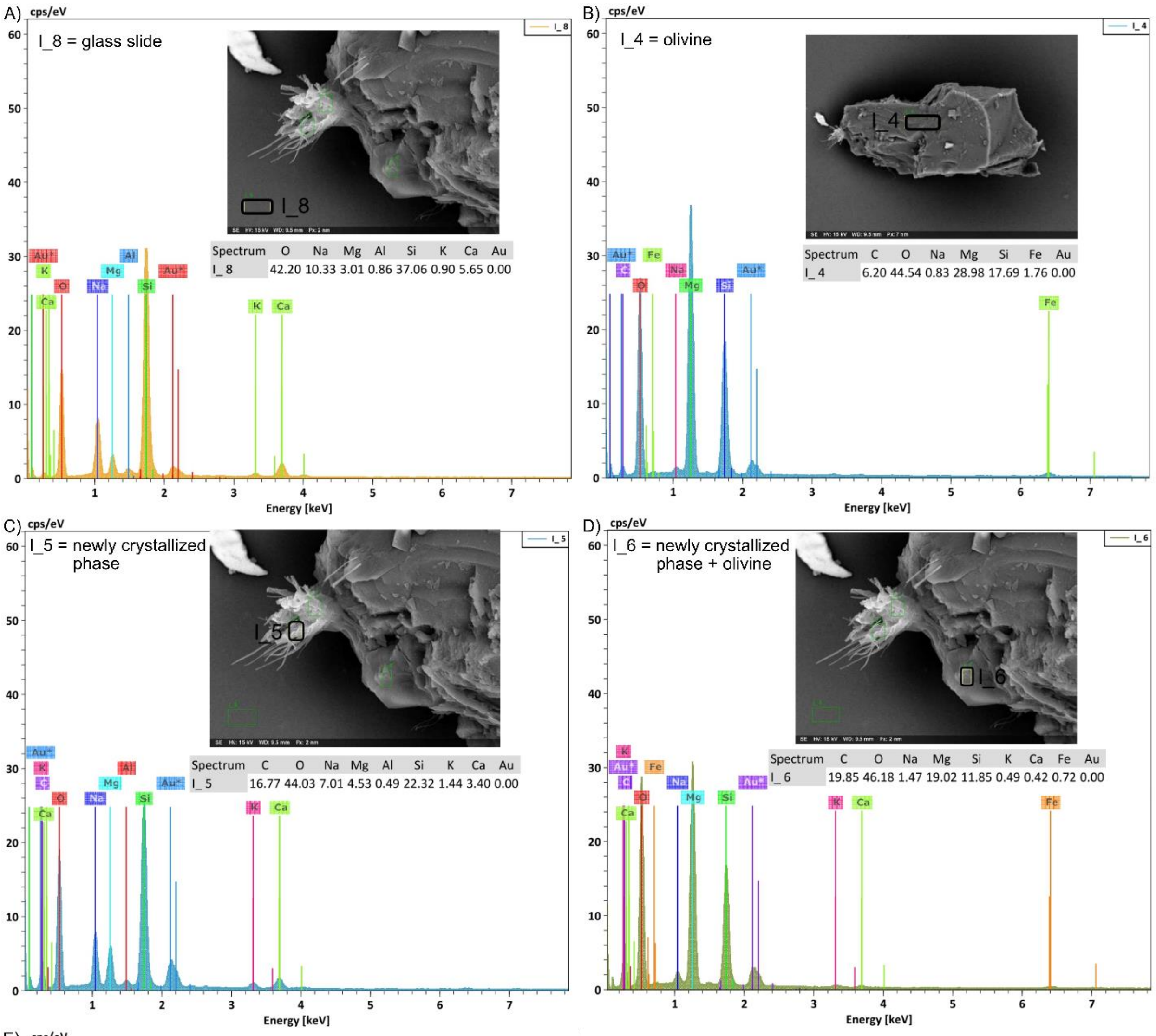

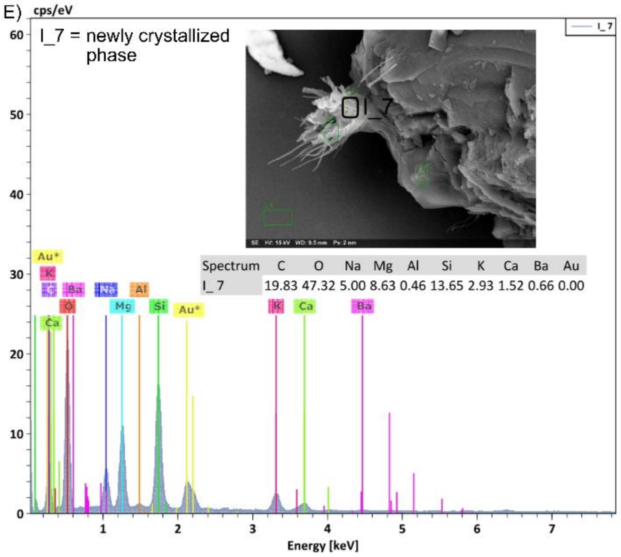

Fig. S2. EDS analyses of the new mineral phase crystallized during fluid escape from an olivine grain. In **(A)** we checked the composition of the glass slide underneath the grain, and in **(B)** we checked the composition of the host olivine. **(C)**, **(D)** and **(E)** present spectra from the newly crystallized phase(s). Sodium and Ca are interpreted to be (largely or entirely) from the glass slide. Most of the Mg and all of the Si are probably from the host olivine. The Au peaks are from the conductive coating and are not considered in the results. The EDS analyses were conducted without standard and, as a consequence, are only semi-quantitative.

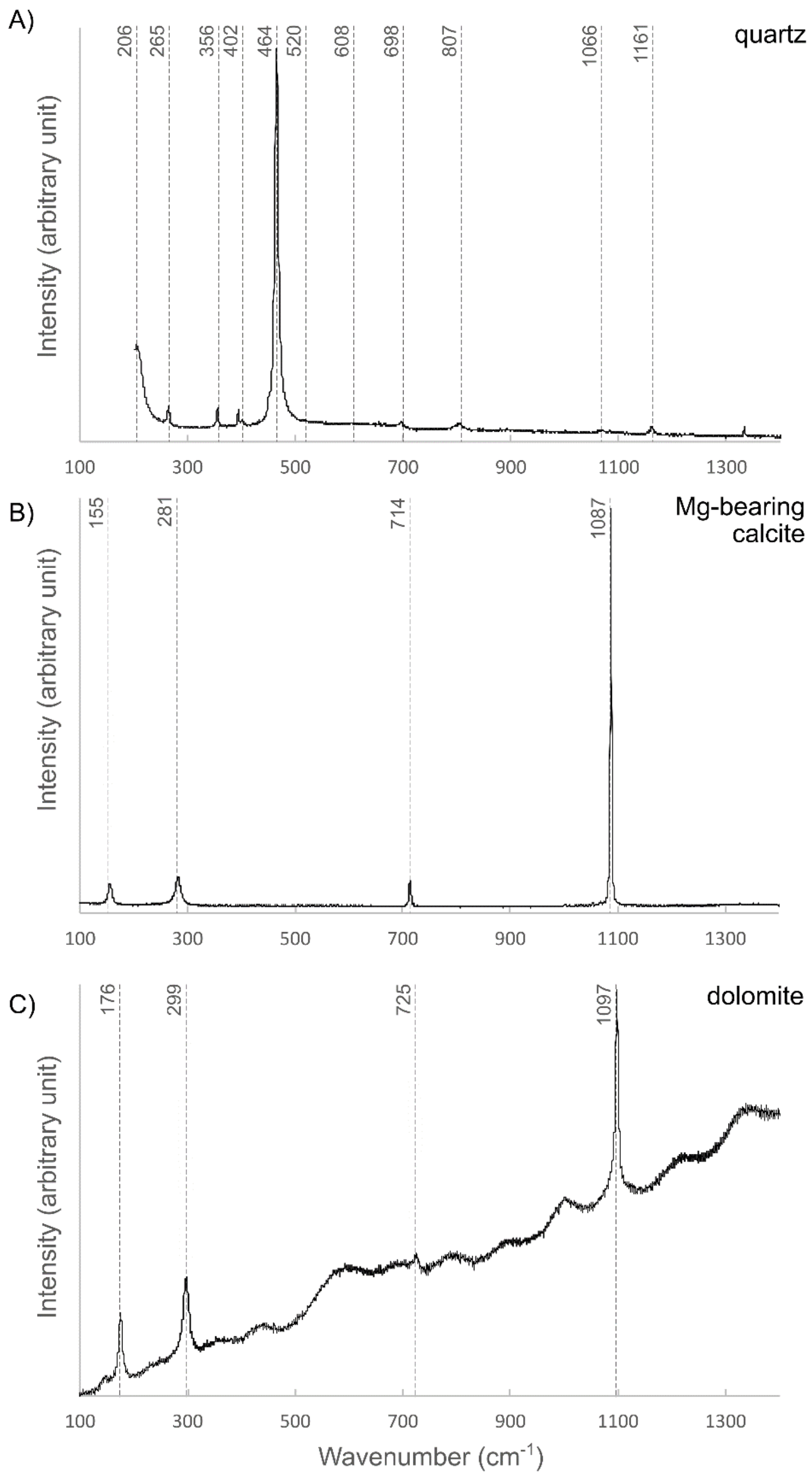

Fig. S3. Raman spectra of silica phase and carbonate minerals included in diamond. Raman spectra of minerals identified as **A)** quartz, **B)** Mg-bearing calcite, and **C)** dolomite. Reference lines for the minerals identified in each spectrum are after Frezzotti et al. (*88*). Spectra are neither smoothed nor baseline corrected.

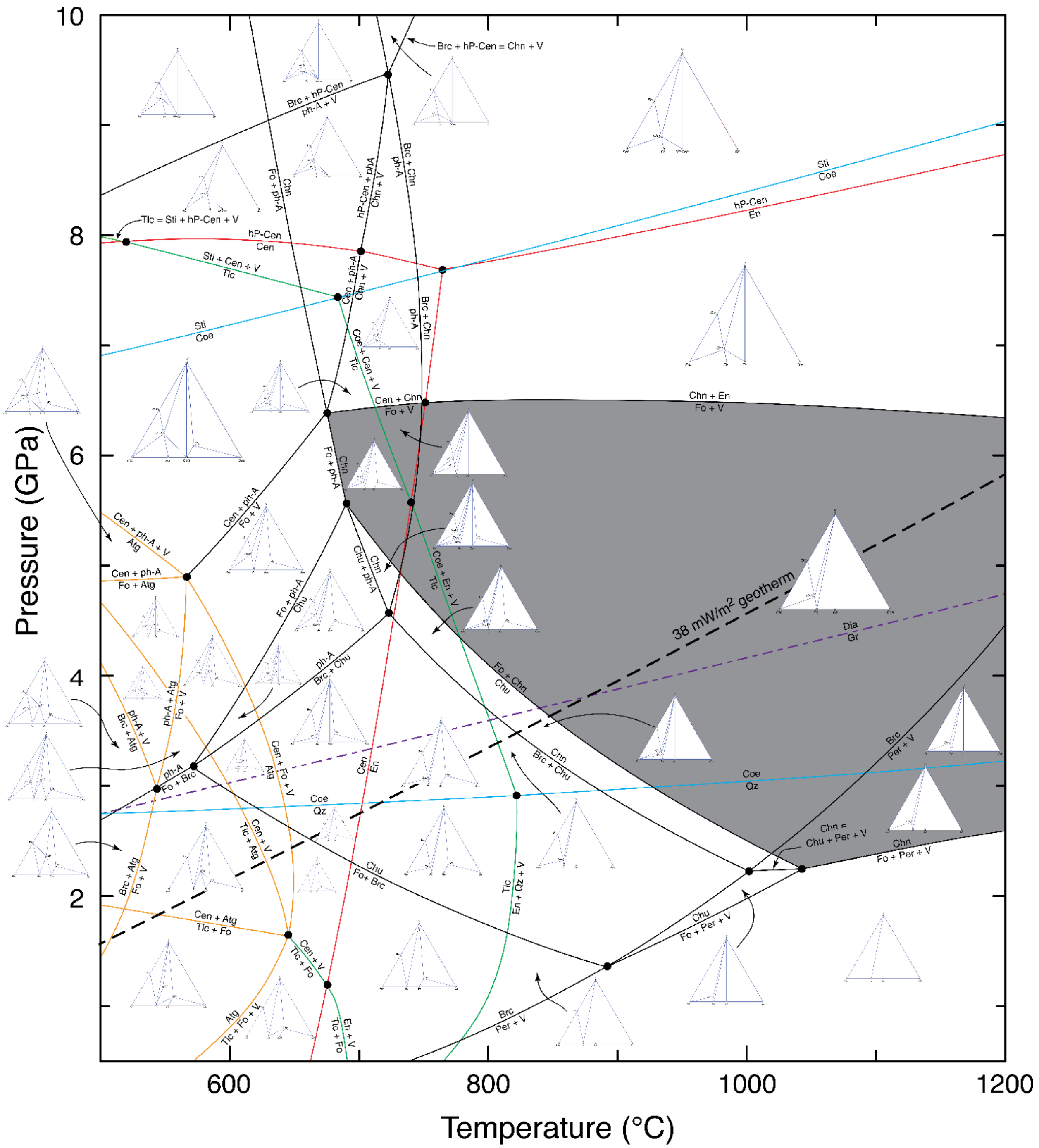

Fig. S4. Phase equilibria in the MgO-SiO$_2$-H$_2$O (MSH) system. P-T projection in the MSH system showing antigorite (orange) and talc (green) breakdown reactions, and the quartz (blue) and enstatite (red) polymorphs transitions. Compatibility triangles for each field are shown. Area highlighted in grey represents the stability field for Fo + Chn + V. Mineral abbreviations (after Warr et al. (*89*)) are as follows: antigorite = Atg; brucite = Brc; chondrodite = Chn; low-P clinoenstatite = Cen; clinohumite = Chu; coesite = Coe; diamond = Dia; enstatite = En; forsterite = Fo; graphite = Gr; periclase = Per; quartz = Qz; stishovite = Sti; talc = Tlc. We used our own abbreviations for high-P clinoenstatite = hP-Cen and phase A = ph-A. A 38mW/m² model geotherm (*71*) and the diamond-graphite boundary (*70*) are shown for reference.

REFERENCES

1. F. Nestola, J. R. Smyth, Diamonds and water in the deep Earth: A new scenario. *Int. Geol. Rev.* **58**, 263–276 (2016).

2. D. G. Pearson, F. E. Brenker, F. Nestola, J. McNeill, L. Nasdala, M. T. Hutchison, S. Matveev, K. Mather, G. Silversmit, S. Schmitz, B. Vekemans, L. Vincze, Hydrous mantle transition zone indicated by ringwoodite included within diamond. *Nature* **507**, 221–224 (2014).

3. J. R. Smyth, β-Mg_2SiO_4: A potential host for water in the mantle? *Am. Min.* **72**, 1051–1055 (1987).

4. M. Faccenda, Water in the slab: A trilogy. *Tectonophysics* **614**, 1–30 (2014).

5. A. B. Thompson, Water in the Earth's upper mantle. *Nature* **358**, 295–302 (1992).

6. M. W. Schmidt, S. Poli, Devolatilization during subduction, in *Treatise on Geochemistry (Second Edition)*, H. D. Holland, K. K. Turekian, Eds. (Elsevier, 2014), pp. 669–701.

7. J. A. Vance, M. A. Dungan, Formation of peridotites by deserpentinization in the Darrington and Sultan areas, Cascade Mountains, Washington, *Geol. Soc. Am. Bull.* **88**, 1497 (1977).

8. P. Ulmer, V. Trommsdorff, Serpentine stability to mantle depths and subduction-related magmatism. *Science* **268**, 858–861 (1995).

9. D. J. Frost, The stability of hydrous mantle phases. *Rev. Mineral. Geochem.* **62**, 243–271 (2006).

10. S-I. Akimoto, K. Yamamoto, K. Aoki, Hydroxyl-clinohumite and hydroxyl-chondrodite: Possible H_2O-bearing minerals in the upper mantle, in *High-Pressure Research*, M.H. Manghnani, S-I. Akimoto, Eds. (Academic Press, 1977), pp. 163–172.

11. P. H. Ribbe, G. V. Gibbs, N. W. Jones, Cation and anion substitutions in the humite minerals. *Mineral. Mag. J. Mineral. Soc.* **36**, 966–975 (1968).

12. N. W. Jones, P. H. Ribbe, G. V. Gibbs, Crystal chemistry of the humite minerals. *Am. Min.* **54**, 391–411 (1969).

13. J. M. Guotana, T. Morishita, I. Nishio, A. Tamura, T. Mizukami, K. Tani, Y. Harigane, K. Szilas, D. G. Pearson, Deserpentinization and high-pressure (eclogite-facies) metamorphic features in the Eoarchean ultramafic body from Isua, Greenland, *Geosci. Front.* **13**, 101298 (2022).

14. I. V. Pekov, E. I. Gerasimova, N. V. Chukanov, Yu. K. Kabalov, N. V. Zubkova, A. E. Zadov, V. O. Yapaskurt, V. M. Gekimyants, D. Yu. Pushcharovskii, Hydroxylchondrodite $Mg_5(SiO_4)_2(OH)_2$: A new mineral of the humite group and its crystal structure. *Doklady Earth. Sci.* **436**, 230–236 (2011).

15. K. Aoki, K. Fujino, M. Akaogi, Titanochondrodite and titanoclinohumite derived from the upper mantle in the Buell Park Kimberlite, Arizona, USA, *USA. Contrib. Mineral. Petrol.* **56**, 243–253 (1976).

16. D. Smith, Titanochondrodite and titanoclinohumite derived from the upper mantle in the Buell Park kimberlite, Arizona, USA, *Contrib. Mineral. Petrol.* **61**, 213–215 (1977).

17. A. Abersteiner, V. S. Kamenetsky, M. Kamenetsky, K. Goemann, K. Ehrig, T. Rodemann, Significance of halogens (F, Cl) in kimberlite melts: Insights from mineralogy and melt inclusions in the Roger pipe (Ekati, Canada). *Chem. Geol.* **478**, 148–163 (2018).

18. A. V. Golovin, V. V. Sharygin, N. P. Pokhilenko, Melt inclusions in olivine phenocrysts in unaltered kimberlites from the Udachnaya-East pipe, Yakutia: Some aspects of kimberlite magma evolution during late crystallization stages. *Petrology* **15**, 168–183 (2007).

19. R. H. Mitchell, Manganoan magnesian ilmenite and titanian clinohumite from the Jacupiranga carbonatite, Sao Paulo Brazil, *Am. Min.* **63**, 544–547 (1978).

20. A. M. Logvinova, R. Wirth, A. A. Tomilenko, V. P. Afanas’ev, N. V. Sobolev, The phase composition of crystal-fluid nanoinclusions in alluvial diamonds in the northeastern Siberian Platform. *Russ. Geol. Geophys.* **52**, 1286–1297 (2011).

21. A. M. Logvinova, I. S. Sharygin, Second natural occurrence of $KFeS_2$ (Hanswilkeite): An inclusion in diamond from the Udachnaya kimberlite pipe (Siberian Craton, Yakutia). *Minerals* **13**, 874 (2023).

22. A. Abersteiner, V. S. Kamenetsky, K. Goemann, A. V. Golovin, I. S. Sharygin, A. Giuliani, T. Rodemann, Z. V. Spetsius, M. Kamenetsky, Djerfisherite in kimberlites and their xenoliths: Implications for kimberlite melt evolution. *Contrib. Mineral. Petrol.* **174**, 8 (2019).

23. R. L. Frost, S. J. Palmer, J. M. Bouzaid, B. J. Reddy, A Raman spectroscopic study of humite minerals. *J. Raman Spectrosc.* **38**, 68–77 (2007).

24. F. R. Boyd, Compositional distinction between oceanic and cratonic lithosphere. *Earth Planet. Sci. Lett.* **96**, 15–26 (1989).

25. T. Stachel, J. W. Harris, The origin of cratonic diamonds; constraints from mineral inclusions. *Ore Geol. Rev.* **34**, 5–32 (2008).

26. A. L. Jaques, A. E. Hall, J. W. Sheraton, C. B. Smith, S. S. Sun, R. M. Drew, C. Foudoulis, K. Ellingsen, Composition of crystalline inclusions and C-isotopic composition of Argyle and Ellendale diamonds, in *Kimberlites and Related Rocks*, J. Ross, A. L. Jaques, J. Ferguson, D. H. Green, O. S. Y. Reilly, R. V Danchin, A. J. A. Janse, Eds. (Geological Society of Australia, 1989), vols. 14; 2, pp. 966–989.

27. T. Stachel, Diamond Inclusion Database. Borealis. V2 (2021); https://doi.org/10.7939/DVN/EJUE1G.

28. F. Nestola, M. E. Regier, R. W. Luth, D. G. Pearson, T. Stachel, C. McCammon, M. D. Wenz, S. D. Jacobsen, C. Anzolini, L. Bindi, J. W. Harris, Extreme redox variations in a superdeep diamond from a subducted slab. *Nature* **613**, 85–89 (2023).

29. R. M. Davies, W. L. Griffin, S. Y. O'Reilly, B. J. Doyle, Mineral inclusions and geochemical characteristics of microdiamonds from the DO27, A154, A21, A418, DO18, DD17 and Ranch Lake kimberlites at Lac de Gras, Slave Craton, Canada. *Lithos* **77**, 39–55 (2004).

30. T. Stachel, J. W. Harris, G. P. Brey, W. Joswig, Kankan diamonds (Guinea) II: Lower mantle inclusion parageneses. *Contrib. Mineral. Petrol.* **140**, 16–27 (2000).

31. J. M. González-Jiménez, G. Plissart, L. N. Garrido, J. A. Padrón-Navarta, T. Aiglsperger, R. Romero, C. Marchesi, A. J. Moreno-Abril, M. Reich, F. Barra, D. Morata, Titanian clinohumite and chondrodite

in antigorite serpentinites from Central Chile: Evidence for deep and cold subduction. *Eur. J. Mineral.* **29**, 959–970 (2018).

32. K. H. Hattori, S. Guillot, Geochemical character of serpentinites associated with high- to ultrahigh-pressure metamorphic rocks in the Alps, Cuba, and the Himalayas: Recycling of elements in subduction zones. *Geochem. Geophys. Geosyst.* **8**, Q09010 (2007)

33. P. Luoni, G. Rebay, M. I. Spalla, D. Zanoni, UHP Ti-chondrodite in the Zermatt-Saas serpentinite: Constraints on a new tectonic scenario. *Am. Min.* **103**, 1002–1005 (2018).

34. T. Shen, J. Hermann, L. Zhang, Z. Lü, J. A. Padrón-Navarta, B. Xia, T. Bader, UHP metamorphism documented in Ti-chondrodite- and Ti-clinohumite-bearing serpentinized ultramafic rocks from Chinese Southwestern Tianshan. *J. Petrol.* **56**, 1425–1458 (2015).

35. B. R. Frost, On the stability of sulfides, oxides, and native metals in serpentinite. *J. Petrol.* **26**, 31–63 (1985).

36. B. W. Evans, Control of the products of serpentinization by the $Fe^{2+}Mg^{-1}$ exchange potential of olivine and orthopyroxene. *J. Petrol.* **49**, 1873–1887 (2008).

37. B. Wunder, W. Schreyer, Antigorite: High-pressure stability in the system MgO-SiO_2-H_2O (MSH). *Lithos* **41**, 213–227 (1997).

38. M. Akaogi, S-I. Akimoto, High-pressure stability of a dense hydrous magnesian silicate $Mg_{23}Si_8O_{42}H_6$ and some geophysical implications. *J. Geophys. Res.* **85**, 6944–6948 (1980).

39. B. Wunder, Equilibrium experiments in the system MgO-SiO_2-H_2O (MSH): Stability fields of clinohumite-OH [$Mg_9Si_4O_{16}$ $(OH)_2$], chondrodite-OH [$Mg_5Si_2O_8$ $(OH)_2$] and phase A ($Mg_7Si_2O_8(OH)_6$). *Contrib. Mineral. Petrol.* 132, 111–120 (1998).

40. K. Yamamoto, S-I. Akimoto, The system MgO-SiO_2-H_2O at high pressures and temperatures; stability field for hydroxyl-chondrodite, hydroxyl-clinohumite and 10 A o-phase. *Am. J. Sci.* **277**, 288–312 (1977).

41. S. Flemetakis, C. Tiraboschi, A. Rohrbach, J. Berndt, S. Klemme, The stability of antigorite in subduction zones revisited: The effect of F on antigorite stability and its breakdown reactions at high pressures and high temperatures, with implications for the geochemical cycles of halogens. *Contrib. Mineral. Petrol.* **177**, 70 (2022).

42. T. John, M. Scambelluri, M. Frische, J. D. Barnes, W. Bach, Dehydration of subducting serpentinite: Implications for halogen mobility in subduction zones and the deep halogen cycle. *Earth Planet. Sci. Lett.* **308**, 65–76 (2011).

43. M. Scambelluri, E. Cannaò, M. Gilio, The water and fluid-mobile element cycles during serpentinite subduction. A review. *A review. Eur. J. Mineral.* **31**, 405–428 (2019).

44. G. Segee-Wright, J. D. Barnes, J. C. Lassiter, D. J. Holmes, G. M. Beaudoin, R. Chatterjee, D. F. Stockli, J. E. Hoffmann, T. John, Halogen enrichment in the North American lithospheric mantle from the dehydration of the Farallon plate. *Geochim. Cosmochim. Acta* **348**, 187–205 (2023).

45. W. Bach, C. J. Garrido, H. Paulick, J. Harvey, M. Rosner, Seawater-peridotite interactions: First insights from ODP Leg 209, MAR 15°N. *Geochem. Geophys. Geosyst.* **5**, Q09F26 (2004).

46. B. W. Evans, K. Hattori, A. Baronnet, Serpentinite: What, why, where? *Elements* **9**, 99–106 (2013).

47. R. T. Gregory, H. P. Taylor, An oxygen isotope profile in a section of Cretaceous oceanic crust, Samail Ophiolite, Oman: Evidence for δ^{18}O buffering of the oceans by deep (>5 km) seawater-hydrothermal circulation at mid-ocean ridges. *J. Geophys. Res. Solid Earth* **86**, 2737–2755 (1981).

48. M. Johnson, M. A. Dungan, J. A. Vance, Stable isotope compositions of olivine and dolomite in peridotites formed by deserpentinization, Darrington area, North Cascades, Washington, *Geochim. Cosmochim. Acta* **41**, 431–435 (1977).

49. A. P. Nutman, M. R. Scicchitano, C. R. L. Friend, V. C. Bennett, A. R. Chivas, Isua (Greenland) ~3700 Ma meta-serpentinite olivine Mg# and δ^{18}O signatures show connection between the early mantle and hydrosphere: Geodynamic implications. *Precambrian Res.* **361**, 106249 (2021).

50. D. Mattey, D. Lowry, C. Macpherson, Oxygen isotope composition of mantle peridotite. *Earth Planet. Sci. Lett.* **128**, 231–241 (1994).

51. M. Regier, A. Miškovic, R. B. Ickert, D. G. Pearson, T. Stachel, R. A. Stern, M. Kopylova, An oxygen isotope test for the origin of Archean mantle roots. *Geochem. Perspect. Lett.* **9**, 6–10 (2018).

52. R. B. Ickert, T. Stachel, R. A. Stern, J. W. Harris, Diamond from recycled crustal carbon documented by coupled $\delta^{18}O$–$\delta^{13}C$ measurements of diamonds and theirinclusions. *Earth Planet. Sci. Lett.* **364**, 85–97 (2013).

53. N. M. Korolev, A. E. Melnik, X.-H. Li, S. G. Skublov, The oxygen isotope composition of mantle eclogites as a proxy of their origin and evolution: A review. *Earth Sci. Rev.* **185**, 288–300 (2018).

54. D. J. Schulze, B. Harte, J. W. Valley, J. M. Brenan, D. M. De R. Channer, Extreme crustal oxygen isotope signatures preserved in coesite in diamond. *Nature* **423**, 68–70 (2003).

55. S. E. Kesson, A. E. Ringwood, Slab-mantle interactions; III, The genesis of diamonds, in *International Congress of Geochemistry and Cosmochemistry*, Y. Bottinga, Ed. (Elsevier, 1988), vols. 70; 1–2, p. 52.

56. K. Li, L. Li, D. G. Pearson, T. Stachel, Diamond isotope compositions indicate altered igneous oceanic crust dominates deep carbon recycling. *Earth Planet. Sci. Lett.* **516**, 190–201 (2019).

57. S. Aulbach, D. E. Jacob, Major- and trace-elements in cratonic mantle eclogites and pyroxenites reveal heterogeneous sources and metamorphic processing of low-pressure protoliths. *Lithos* **262**, 586–605 (2016).

58. H. H. Helmstaedt, D. J. Schulze, Southern African kimberlites and their mantle sample: Implications for Archean tectonics and lithosphere evolution, in *Kimberlites and Related Rocks*, J. Ross, Ed. (Blackwell, 1989), pp. 358–368.

59. D. E. Jacob, Nature and origin of eclogite xenoliths from kimberlites. *Lithos* **77**, 295–316 (2004).

60. T. Stachel, S. Aulbach, J. W. Harris, Mineral Inclusions in lithospheric diamonds. *Rev. Mineral. Geochem.* **88**, 307–391 (2022).

61. F. R. Boyd, J. J. Gurney, Diamonds and the African lithosphere. *Science* **232**, 472–477 (1986).

62. D. G. Pearson, J. M. Scott, J. Liu, A. Schaeffer, L. H. Wang, J. van Hunen, K. Szilas, T. Chacko, P. B. Kelemen, Deep continental roots and cratons. *Nature* **596**, 199–210 (2021).

63. M. J. Walter, Melting residues of fertile peridotite and the origin of cratonic lithosphere, in *Mantle Petrology: Field Observations and High Pressure Experimentation: A Tribute to Francis R. (Joe) Boyd*, Y. Fei, C. M. Bertka, B. O. Mysen, Eds. (The Geochemical Society, 1999), vol. 6, pp. 225–239.

64. J. J. Gurney, A correlation between garnets and diamonds in kimberlites, in *Kimberlite Occurrence and Origin: A Basis for Conceptual Models in Exploration*, J. E. Glover, P. G. Harris, Eds. (University of Western Australia, 1984), vol. 8, pp. 143–166.

65. D. J. Schulze, Do peridotite-suite diamonds form in subducted serpentinites?, in *Fourth International Kimberlite Conference*, C. B. Smith, Ed. (Geological Society of Australia, 1986), vol. 16, pp. 424–425.

66. R. M. Davies, W. L. Griffin, S. Y. O'Reilly, A. S. Andrew, Unusual mineral inclusions and carbon isotopes of alluvial diamonds from Bingara, eastern Australia. *Lithos* **69**, 51–66 (2003).

67. J. W. Harris, K. V. Smit, Y. Fedortchouk, M. Moore, Morphology of monocrystalline diamond and its inclusions. *Rev. Mineral. Geochem.* **88**, 119–166 (2022).

68. S. Arai, Contact metamorphosed dunite-harzburgite complex in the Chugoku district, western Japan. *Contrib. Mineral. Petrol.* **52**, 1–16 (1975).

69. Y. Chen, K. Ye, S. Guo, T.-F. Wu, J.-B. Liu, Multistage metamorphism of garnet orthopyroxenites from the Maowu mafic–ultramafic complex, Dabieshan UHP terrane, eastern China. *Int. Geol. Rev.* **55**, 1239–1260 (2013).

70. H. W. Day, A revised diamond-graphite transition curve. *Am. Min.* **97**, 52–62 (2012).

71. D. Hasterok, D. S. Chapman, Heat production and geotherms for the continental lithosphere. *Earth Planet. Sci. Lett.* **307**, 59–70 (2011).

72. T. R. McGetchin, L. T. Silver, A. A. Chodos, Titanoclinohumite: A possible mineralogical site for water in the upper mantle. *J. Geophys. Res.* **75**, 255–259 (1970).

73. T. Komabayashi, S. Omori, S. Maruyama, Experimental and theoretical study of stability of dense hydrous magnesium silicates in the deep upper mantle. *Phys. Earth Planet. Inter.* **153**, 191–209 (2005).

74. K. Yamamoto, S.-I. Akimoto, High pressure and high temperature investigations in the system MgO SiO_2 H_2O. *J. Solid State Chem.* **9**, 187–195 (1974).

75. V. M. Gekimyants, E. V. Sokolova, E. M. Spiridonov, G. Ferraris, N. V. Chukanov, M. Prencipe, V. N. Avdonin, Yu. A. Polenov, Hydroxylclinohumite $Mg_9(SiO_4)_4(OH,F)_2$—A new mineral of the humite group. *Proc. Russ. Mineral. Soc.* **128**, 64–70 (1999).

76. T. J. B. Holland, R. Powell, An improved and extended internally consistent thermodynamic dataset for phases of petrological interest, involving a new equation of state for solids. *J. Metam. Geol.* **29**, 333–383 (2011).

77. L. Hughes, A. Pawley, Fluorine partitioning between humite-group minerals and aqueous fluids: Implications for volatile storage in the upper mantle. *Contrib. Mineral. Petrol.* **174**, 78 (2019).

78. T. Stachel, R. W. Luth, Diamond formation: Where, when and how? *Lithos* **220-223**, 200–220 (2015).

79. Y. Bussweiler, G. P. Brey, D. G. Pearson, T. Stachel, R. A. Stern, M. F. Hardman, B. A. Kjarsgaard, S. E. Jackson, The aluminum-in-olivine thermometer for mantle peridotites—Experimental versus empirical calibration and potential applications. *Lithos* **272–273**, 301–314 (2017).

80. J. C. M. De Hoog, L. Gall, D. H. Cornell, Trace-element geochemistry of mantle olivine and application to mantle petrogenesis and geothermobarometry. *Chem. Geol.* **270**, 196–215 (2010).

81. C. G. Ryan, W. L. Griffin, N. J. Pearson, Garnet geotherms: Pressure-temperature data from Cr-pyrope garnet xenocrysts in volcanic rocks. *J. Geophys. Res. Solid Earth* **101**, 5611–5625 (1996).

82. M. W. Schmidt, P. Ulmer, A rocking multianvil: Elimination of chemical segregation in fluid-saturated high-pressure experiments. *Geochim. Cosmochim. Acta* **68**, 1889–1899 (2004).

83. S. Aulbach, T. Stachel, L. M. Heaman, J. A. Carlson, Microxenoliths from the Slave Craton: Archives of diamond formation along fluid conduits. *Lithos* **126**, 419–434 (2011).

84. T. Stachel, J. W. Harris, L. Hunt, K. Muehlenbachs, A. Kobussen, EIMF, Argyle diamonds: How subduction along the Kimberley craton edge generated the world's biggest diamond deposit, in *Geoscience and Exploration of the Argyle, Bunder, Diavik, and Murowa Diamond Deposits*, Andy T. Davy, Chris B. Smith, Herwart Helmstaedt, A. Lynton Jaques, John J. Gurney, Eds. (Society of Economic Geologists, 2018), vol. 20.

85. J. T. Armstrong, CITZAF: A package of correction programs for the quantitative electron micro beam x-ray analysis of thick polished materials, thin films, and particles. *Microbeam Anal.* **4**, 177–200 (1995).

86. R. B. Ickert, R. A. Stern, Matrix corrections and error analysis in high-precision SIMS $^{18}O/^{16}O$ measurements of Ca–Mg–Fe garnet. *Geostand. Geoanal. Res.* **37**, 429–448 (2013).

87. K. E. Kuebler, B. L. Jolliff, A. Wang, L. A. Haskin, Extracting olivine (Fo–Fa) compositions from Raman spectral peak positions. *Geochim. Cosmochim. Acta* **70**, 6201–6222 (2006).

88. M. L. Frezzotti, F. Tecce, A. Casagli, Raman spectroscopy for fluid inclusion analysis. *J. Geochem. Explor.* **112**, 1–20 (2012).

89. L. N. Warr, IMA–CNMNC approved mineral symbols. *Mineral. Mag.* **85**, 291–320 (2021).
